# Fabrication of Advanced Cellulosic Triboelectric Materials via Dielectric Modulation

**DOI:** 10.1002/advs.202206243

**Published:** 2023-03-26

**Authors:** Guoli Du, Jinlong Wang, Yanhua Liu, Jinxia Yuan, Tao Liu, Chenchen Cai, Bin Luo, Siqiyuan Zhu, Zhiting Wei, Shuangfei Wang, Shuangxi Nie

**Affiliations:** ^1^ School of Light Industry and Food Engineering Guangxi University Nanning 530004 P. R. China

**Keywords:** cellulose, charge density, dielectric, nanogenerators, triboelectric materials

## Abstract

The rapid rise of triboelectric nanogenerators (TENGs), which are emerging energy conversion devices in advanced electronics and wearable sensing systems, has elevated the interest in high‐performance and multifunctional triboelectric materials. Among them, cellulosic materials, affording high efficiency, biodegradability, and customizability, are becoming a new front‐runner. The inherently low dielectric constant limits the increase in the surface charge density. However, owing to its unique structure and excellent processability, cellulose shows great potential for dielectric modulation, providing a strong impetus for its advanced applications in the era of Internet of Things and artificial intelligence. This review aims to provide comprehensive insights into the fabrication of dielectric‐enhanced cellulosic triboelectric materials via dielectric modulation. The exceptional advantages and research progress in cellulosic materials are highlighted. The effects of the dielectric constant, polarization, and percolation threshold on the charge density are systematically investigated, providing a theoretical basis for cellulose dielectric modulation. Typical dielectric characterization methods are introduced, and their technical characteristics are analyzed. Furthermore, the performance enhancements of cellulosic triboelectric materials endowed by dielectric modulation, including more efficient energy harvesting, high‐performance wearable electronics, and impedance matching via material strategies, are introduced. Finally, the challenges and future opportunities for cellulose dielectric modulation are summarized.

## Graphical abstract

In this review, the unique advantages and research progress of cellulosic triboelectric materials are summarized. The effects of dielectric constant, polarization, and percolation threshold on the surface charge density of cellulosic triboelectric materials are systematically elaborated. Comprehensive insights into the construction of high‐performance and multifunctional advanced cellulosic triboelectric materials via dielectric modulation are provided.

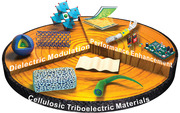

## 1 Introduction

Cellulose is one of the oldest natural polymers on Earth; it has a unique structure and special properties.^[^ ^1^ ^]^ Cellulose has been used for centuries as a key material in various practical applications and has contributed to human evolution and technological developments.^[^ ^2^ ^]^ With the rapid development of the Fourth Industrial Revolution, several requirements have been put forward for modern advanced materials in regard to convenience and intelligence.^[^ ^3^ ^]^ Cellulose is a renewable, low‐cost, and biodegradable natural biomass material, with promising properties: suitable mechanical properties, easy processability, dielectric property, piezoelectricity, and convertibility.^[^ ^4^ ^]^ Additionally, nanocellulose has offered great advantages in the synthesis of advanced functional materials owing to its excellent properties and unique shape. Hence, cellulose shows much adaptability and is highly competitive among many polymer materials that have been widely used in cutting‐edge technology fields, such as renewable energy, environmental protection, biomedicine, aerospace, advanced electronic equipment, and green chemistry.^[^ ^5^ ^]^

Among prospective cellulose‐based materials, cellulosic triboelectric materials have received extensive attention owing to their percolation energy harvesting and conversion capabilities.^[^ ^6^ ^]^ In 2012, Wang et al.^[^ ^7^ ^]^ developed a triboelectric nanogenerator (TENG) capable of converting mechanical energy into electrical energy. TENG has now proven to be a reliable technology for green energy harvesting and conversion because of its low cost, multiple structures, high fabrication efficiency, excellent environmental adaptability, and stable output.^[^ ^8^ ^]^ The fundamental of TENGs is the coupling effect of contact electrification (CE) and electrostatic induction caused by the periodic contact and separation of triboelectric materials, respectively.^[^ ^9^ ^]^ As the main component for CE, triboelectric materials directly affect the energy conversion efficiency and application prospects of TENGs; hence, they have become a trending research topic in recent years.^[^ ^10^ ^]^ A large number of lone electron pairs of oxygen atoms endows cellulose with extremely high electron‐donating ability, i.e., the tendency to lose electrons, making cellulose a promising triboelectric material.^[^ ^11^ ^]^ As the most abundant biomass resource on Earth, the sustainability of cellulose enables it to meet the needs of the Internet of Things (IoT) for large‐scale self‐powered sensing arrays.^[^ ^12^ ^]^ Additionally, the inherent biodegradability and biocompatibility of cellulose render it environmentally friendly and harmless to human health, unlike most petroleum‐based polymer triboelectric materials.^[^ ^13^ ^]^ In addition, the excellent processability enables the customization of cellulose into triboelectric materials with different properties, shapes, and sizes to suit specific environmental and functional requirements.^[^ ^14^ ^]^ This is highly instrumental in expanding the application prospects of TENGs. To date, research on cellulosic triboelectric materials has made great progress, including simple paper‐based, wood‐based, and gel‐structured triboelectric materials, as well as chemical functionalization of the surface of cellulosic materials. Numerous efforts have been made to improve the triboelectric properties of cellulosic triboelectric materials.^[^ ^15^ ^]^ However, much research is needed to achieve advanced cellulosic triboelectric materials toward completely harnessing the great potential of cellulose.

A large number of research teams have proposed many methods to enhance and improve the output performance of TENGs, such as charge excitation of TENG,^[^ ^16^ ^]^ structure optimization and innovation,^[^ ^17^ ^]^ circuit design and management,^[^ ^18^ ^]^ etc. These methods also play a positive role in expanding the application prospects of TENGs. In the choice of materials, the surface charge density of triboelectric materials determines the output performance of TENGs.^[^ ^19^ ^]^ Based on dielectric theory, the dielectric constant (*k*) is the ability of a material to polarize in an alternating electric field.^[^ ^20^ ^]^ It also reflects the ability of triboelectric materials to generate and retain triboelectric charges during the working process.^[^ ^21^ ^]^ According to the frequency range, the polarization types can be divided into electronic polarization, ionic polarization, dipole polarization, and interface polarization. Most polymeric materials are composed of covalent bonds such as C—H that do not induce polarization, so their inherently low dielectric constants largely limit the surface charge density.^[^ ^20^ ^]^ Therefore, dielectric modulation is typically performed to improve the surface charge density of triboelectric materials by enhancing their polarization response, thereby fundamentally increasing their effective dielectric constant.^[^ ^22^ ^]^ Cellulose has a high capability of dielectric modulation because of its triboelectric properties and wide availability. In addition, owing to its unique structure and processability, cellulose has great potential for dielectric modulation via advanced material design strategies and processing technologies.^[^ ^23^ ^]^ In recent years, numerous effective modulation methods have been proposed, such as chain‐structure modification,^[^ ^24^ ^]^ polymer blending,^[^ ^25^ ^]^ and conductive‐ or high‐k‐filler doping.^[^ ^26^ ^]^ These direct approaches increase the dielectric constant and surface charge density, boosting the development of cellulosic triboelectric materials. Owing to dielectric modulation, the application of cellulose‐based TENGs has been significantly broadened from self‐powered multifunctional sensing to wearable health care systems,^[^ ^27^ ^]^ microwave devices to electromagnetic interference (EMI) shielding,^[^ ^28^ ^]^ and human‐machine interfaces to intelligent robots.^[^ ^29^ ^]^ High‐k cellulosic triboelectric materials exhibit promising application potential in numerous emerging smart technologies and advanced manufacturing fields.

**Figure 1:**
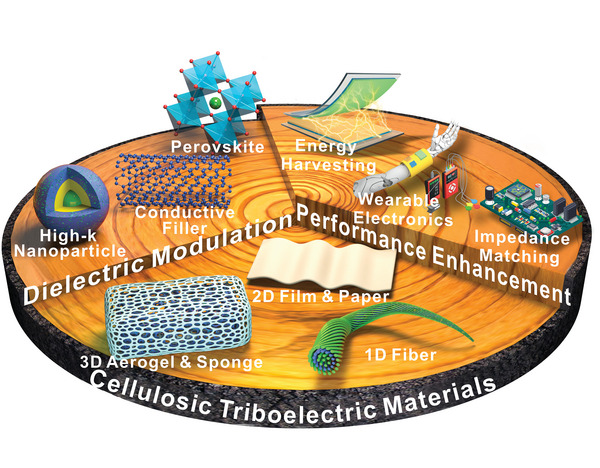
Construction of dielectric‐enhanced cellulosic triboelectric materials with performance enhancement via dielectric modulation.

This review focuses on fabricating dielectric‐enhanced cellulosic triboelectric materials via dielectric modulation. The dielectric properties of cellulosic triboelectric materials are improved by doping with conductive or high‐k fillers, thereby obtaining better triboelectric properties. This modulation method applies to cellulosic triboelectric materials of different dimensions as well as different types of fillers (**Figure** **1**). The excellent properties and unique advantages of high‐k cellulosic triboelectric materials are demonstrated by analyzing the preparation methods of cellulosic triboelectric materials. Furthermore, the principles and strategies of dielectric modulation are elaborated from both theoretical and strategic perspectives. Several commonly used dielectric characterization methods are introduced in detail, and their technical characteristics are analyzed. Subsequently, the performance enhancements endowed by dielectric modulation to cellulosic triboelectric materials are reviewed, including more efficient energy harvesting, high‐performance wearable electronics, and impedance matching via material strategies. Finally, the challenges and prospects are discussed in view of the large‐scale commercialization of advanced high‐k cellulosic triboelectric materials.

## 2 Overview of Cellulosic Triboelectric Materials for Dielectric Modulation

The structure, properties, and preparation methods of triboelectric materials play crucial roles in the performance of TENGs. In this section, the intrinsic advantages, triboelectric properties, and common preparation methods of cellulosic triboelectric materials are introduced, and the potential of enhancing the triboelectric properties of cellulose by dielectric effects is discussed.

### 2.1 Intrinsic Advantages of Cellulosic Materials

**Figure 2:**
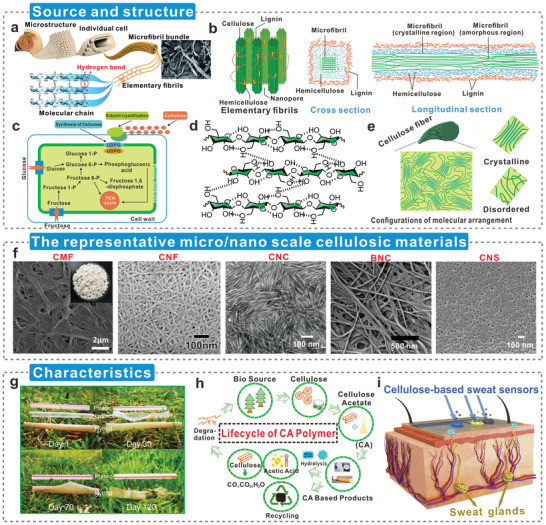
Intrinsic advantages of cellulosic materials. a) Schematic illustration of hierarchical fibril structure and morphologies of wood cellulose fiber. Reproduced with permission.^[^ ^36a^ ^]^ Copyright 2016, American Chemical Society. b) Schematic diagram of lignocellulosic components. Reproduced with permission.^[^ ^44^ ^]^ Copyright 2020, Springer Nature. c) Bacteria biosynthesize bacterial cellulose. Reproduced with permission.^[^ ^42^ ^]^ Copyright 2017, MDPI. d) Cellulose molecular chain. Reproduced with permission.^[^ ^44^ ^]^ Copyright 2020, Springer Nature. e) Crystalline and amorphous regions in cellulose molecules. Reproduced with permission.^[^ ^55^ ^]^ Copyright 2021, Wiley‐VCH. f) The representative micro/nanoscale cellulosic materials. CMF, Reproduced with permission.^[^ ^66^ ^]^ Copyright 2011, Elsevier. CNF, Reproduced with permission.^[^ ^67^ ^]^ Copyright 2007, American Chemical Society. CNC, Reproduced with permission.^[^ ^68^ ^]^ Copyright 2014, Wiley‐VCH. BNC, Reproduced with permission.^[^ ^69^ ^]^ Copyright 2007, American Chemical Society. g) Straws made of cellulosic material degrade rapidly in the natural environment. Reproduced with permission.^[^ ^59^ ^]^ Copyright 2020, Wiley‐VCH. h) Cyclic processes of cellulosic products in ecosystems and human societies. Reproduced with permission.^[^ ^60^ ^]^ Copyright 2021, Elsevier. i) Cellulose‐based self‐powered sweat sensor for human skin with natural biocompatibility. Reproduced with permission.^[^ ^62^ ^]^ Copyright 2022, Wiley‐VCH.

Cellulose is the most abundant renewable biopolymer and is derived from a wide range of sources, including wood,^[^ ^30^ ^]^ plants,^[^ ^31^ ^]^ algae,^[^ ^32^ ^]^ bacteria,^[^ ^33^ ^]^ and tunicates.^[^ ^34^ ^]^ Lignocellulose and bacterial cellulose (BC) are widely used as triboelectric materials owing to their few polymorphic constraints, good processability, and excellent physical and chemical properties.^[^ ^35^ ^]^ Trees have a natural hierarchical structure and are the main source of lignocellulosic triboelectric materials (**Figure** **2**a).^[^ ^36^ ^]^ In trees, lignocellulose is mainly present in the form of complexes in the cell walls of plant cells. It is mainly composed of cellulose, hemicellulose, and lignin.^[^ ^37^ ^]^ Lignin and hemicellulose are closely linked to cellulose and act as protective barriers (Figure 2b).^[^ ^38^ ^]^ To achieve better mechanical properties and more unique uses of materials, it is generally necessary to separate cellulose from composites.^[^ ^39^ ^]^ Mechanical fibrillation is commonly used in industrial production and scientific research, and the primary product is called cellulose microfibrils (CMFs).^[^ ^40^ ^]^ CMFs are the most common cellulose fibers in daily life, widely present in paper products and packaging supplies. To further obtain cellulose fibers of smaller size, i.e., cellulose nanofibers (CNFs) at the nanoscale, chemical pretreatment is usually required to fibrillate the thicker fibers down to elementary fibrils.^[^ ^41^ ^]^ Overall, this is a top‐down process for producing nanocellulose. The difference is that the production of BC is a typical bottom‐up process. BC is produced biosynthetically by nonpathogenic bacteria (Figure 2c),^[^ ^42^ ^]^ such as *Sarcina*, *Rhizobium*, *Komagataeibacter*.^[^ ^43^ ^]^ Since the cellulose produced by bacteria is nanoscale, BC can also be called bacterial nanocellulose (BNC). Cellulose molecules interconnect hundreds of D‐glucopyranosyl groups through *β*‐1,4‐glycosidic bonds (Figure 2d).^[^ ^44^ ^]^ The unique linear configuration arises from hydrogen bonding between the hydroxyl and oxygen atoms of the adjacent glucose groups. The excellent mechanical properties and network structure conferred by these hydrogen bonds are beneficial for charge transport.^[^ ^45^ ^]^ The arrangement of cellulose molecular chains is divided into crystalline regions and amorphous regions according to their regularity. The structure formed by the alternating distribution of regular crystalline and disordered amorphous regions endows cellulose with high flexibility, thermal stability, and mechanical strength (Figure 2e).^[^ ^46^ ^]^ In the production of cellulose nanocrystals (CNCs), the disordered amorphous parts are usually removed in an acid hydrolysis reaction, leaving only highly ordered crystalline regions.^[^ ^47^ ^]^ Therefore, CNCs have higher crystallinity than CNFs. These different cellulose types are all polymorphs of cellulose I. The regenerated cellulose obtained by changing the physical structure becomes cellulose II, which differs from cellulose I in molecular and crystal structure.^[^ ^48^ ^]^ Thus showing different physical and chemical properties. This is an important distinction between cellulosic materials and at the same time their most fascinating property.

In the development and utilization of cellulose, micro/nanoscale cellulosic materials with excellent mechanical strength and physicochemical properties are widely favored.^[^ ^49^ ^]^ They have different characteristics and applications. CMFs are usually produced only by mechanical refining, CNFs with high aspect ratio and specific modulus, CNCs with high crystallinity, BNCs with high purity and water content, and highly homogeneous cellulose nanosheets (CNSs) (Figure 2f). Nanocellulose is generally used as the main raw material for stimuli‐responsive materials, shape‐memory materials, self‐healing materials, and adhesive materials because of its excellent customizability.^[^ ^50^ ^]^ At the same time, cellulosic materials have gradually emerged as promising candidates for many cutting‐edge applications such as nanogenerators,^[^ ^51^ ^]^ electrochemical energy storage,^[^ ^52^ ^]^ optoelectronic devices,^[^ ^53^ ^]^ and flexible electronics.^[^ ^54^ ^]^

The unique molecular arrangement endows cellulosic materials with special properties. The good mechanical performance, flexibility, and toughness of cellulose fiber are attributed to the excellent chain length and abundant intermolecular and intramolecular hydrogen bonds of cellulose.^[^ ^55^ ^]^ It is capable of manufacturing highly flexible cellulosic triboelectric materials (paper, film, elastomer, etc.) for advanced electronic devices such as electronic skin, soft robots, energy storage, and wearable smart devices.^[^ ^56^ ^]^ Additionally, the specific strength of cellulose ranges from 1.0 to 5.1 GPa cm^3^ g^−1^, which is higher than most engineering materials including titanium alloys.^[^ ^57^ ^]^ Such high structural strength ensures the dimensional stability of cellulosic triboelectric materials in self‐powered smart sensing applications. Cellulosic materials with good biodegradability potentially address the environmental problems associated with petroleum‐based polymers.^[^ ^58^ ^]^ Nearly all commonly used plastics are not degradable or chemically reactive in the natural environment. Fortunately, cellulosic materials can degrade rapidly in the natural environment without causing additional burdens on the ecological environment (Figure 2g).^[^ ^59^ ^]^ Moreover, they form a stable and mature circulation system in the ecosystem and human society and maximize economic benefits while protecting the environment (Figure 2h).^[^ ^60^ ^]^ Their biocompatibility and low cytotoxicity contribute to the reliable safety performance of cellulosic medical materials, which have broad development potential in the fields of medical treatment, smart health care, and self‐powered health monitoring.^[^ ^61^ ^]^ Sweat sensors fabricated using CNFs can be directly applied to human skin, providing new opportunities for cellulosic materials in the fields of smart health and electronic skin (Figure 2i).^[^ ^62^ ^]^ Cellulose is derivatizable because of the numerous active hydroxyl groups (—OH) present in the molecular chains.^[^ ^63^ ^]^ By grafting specific functional groups to cellulose molecules, new properties such as hydrophobicity, thermal stability, and high triboelectric polarity can be obtained; the inherent properties are retained. The charged groups on the nanocellulose surface can be stoichiometrically counterion‐exchanged into diverse metal and alkylammonium ions,^[^ ^64^ ^]^ imparting the modified nanocellulose with various new functions, such as catalysis, super deodorization, and gas separation.^[^ ^65^ ^]^ Such versatile cellulosic materials significantly enhance the selectivity of triboelectric materials, providing a myriad of possibilities for future applications of cellulose‐based TENGs.

### 2.2 Triboelectric Properties of Cellulosic Materials

**Figure 3:**
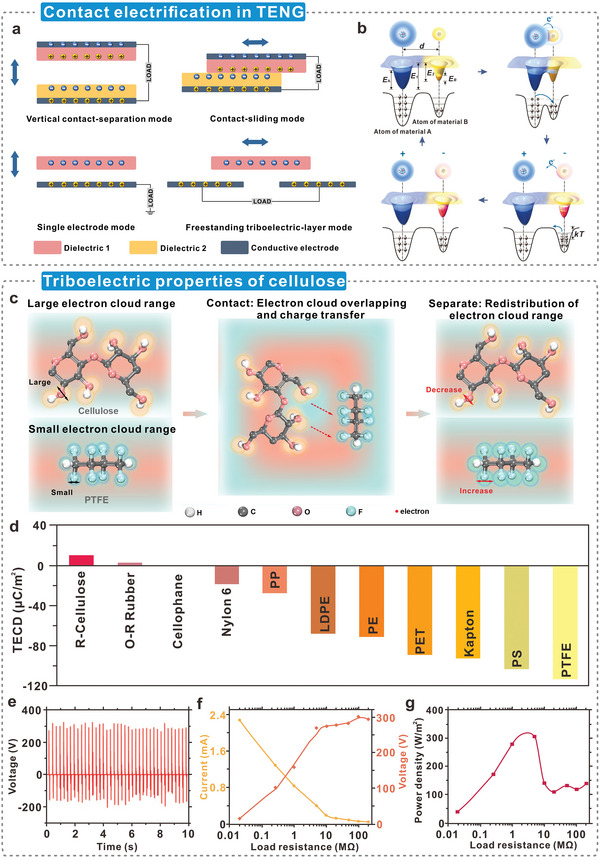
Triboelectric properties of cellulosic materials. a) Four working modes of TENGs. Reproduced with permission.^[^ ^71^ ^]^ Copyright 2021, Wiley‐VCH. b) Electron cloud/potential model used to describe the specific process of contact electrification. Reproduced with permission.^[^ ^74^ ^]^ Copyright 2018, Wiley‐VCH. c) The electron transfer mechanism of cellulose in contact electrification, another triboelectric layer is PTFE as an example. When two materials come into contact, electrons jump from the less electronegative atom to the electron cloud of the more electronegative atom. Reproduced with permission.^[^ ^78^ ^]^ Copyright 2022, Wiley‐VCH. d) Quantified triboelectric series of some common polymers, the triboelectric polarity of cellulose is the opposite of most polymers, and e–g) Output performance of TENG using regenerated cellulose and cellophane as triboelectric materials. Its power density can reach 300 W m^−2^. Reproduced with permission.^[^ ^81^ ^]^ Copyright 2020, Wiley‐VCH.

TENGs are driven by Maxwell's displacement current, by which they can convert the surrounding irregular, low‐frequency, distributed mechanical energy into electrical energy.^[^ ^70^ ^]^ They operate in four different modes, namely, vertical contact–separation (CS) mode, lateral sliding (LS) mode, single electrode (SE) mode, and freestanding triboelectric (FT) layer mode (**Figure** **3**a).^[^ ^71^ ^]^ These working modes differ in their applicable scenarios and conversion efficiencies, but the cellulosic material can effectively function as the tribolayer.^[^ ^72^ ^]^ The surface charge density generated by triboelectric material reflects the triboelectric performance.^[^ ^73^ ^]^ The electron cloud/potential model based on fundamental electron cloud interactions demonstrates the specific CE process in cellulosic triboelectric materials (Figure 3b).^[^ ^74^ ^]^ When two materials come in contact with an external force, the electron clouds overlap to form ionic or covalent bonds. At this point, the potential barrier between the two materials decreases, and the minimum potential energy required for an electron to escape from the material is less than the energy level occupied by the electron; hence, the electron leaps from the atom of one material to an atom of another material.^[^ ^75^ ^]^ The degree and efficiency of electron transfer between materials depend on the “charge affinity” (i.e., the magnitude and direction of the surface potential difference) of the triboelectric materials, which is influenced by various factors, such as dielectric properties, the electronegativity of atoms, the ability to gain or lose electrons, surface roughness, and local topography.^[^ ^76^ ^]^

Owing to the presence of abundant polar hydroxyl groups, cellulose has a strong electron‐donating ability and is considered an efficient positive triboelectric material.^[^ ^77^ ^]^ Figure 3c is used to describe the electron transfer of cellulose during contact electrification.^[^ ^78^ ^]^ The “clouds and fog” in the diagram represent electron clouds around atoms. During the contact between cellulose and polytetrafluoroethylene (PTFE), the electron clouds of the two overlap. Lone electrons from low electronegativity atoms (O) jump to high electronegativity atoms (F), resulting in electron transfer between the two materials.^[^ ^79^ ^]^ Additionally, the surface roughness of cellulosic materials, conferred by the crisscross of fibers, can be further customized via mechanical or chemical treatment to contribute to CE.^[^ ^80^ ^]^ Recently, cellulosic triboelectric materials have attracted considerable research interest. In 2020, Zhang and co‐workers^[^ ^81^ ^]^ conducted triboelectric charge density (TECD) experiments to compare the triboelectric properties of commonly used triboelectric materials (Figure 3d). The results show that the TECD of regenerated cellulose is +10.05 µC m^−2^, which is 3 times that of oil‐resistant nitrile rubber (2.95 µC m^−2^). The TECD of cellophane is +0.17 µC m^−2^, nearly 60 times less than that of regenerated cellulose. The TENG with regenerated cellulose and cellophane as triboelectric materials exhibited excellent output performance (Figure 3e,f), with an output power density of 300 W m^−2^ (Figure 3g). This demonstrates the huge potential for cellulose, upon dielectric modulation, as a high‐performance advanced triboelectric material.

### 2.3 Cellulosic Triboelectric Materials Prepared by Conventional Methods

**Figure 4:**
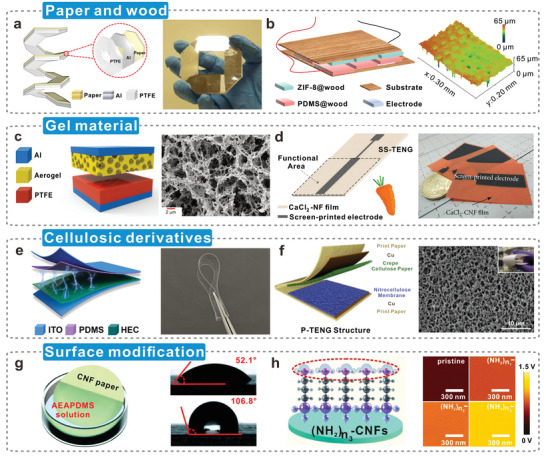
Preparation method of cellulosic triboelectric materials. a) Paper‐based TENG with common raw materials and simple preparation. Reproduced with permission.^[^ ^83^ ^]^ Copyright 2015, American Chemical Society. b) Wood‐based triboelectric materials with tunable triboelectric polarity and rich surface roughness structure. Reproduced with permission.^[^ ^85^ ^]^ Copyright 2021, Elsevier. c) Cellulose II aerogel is used as the triboelectric layer of TENG with abundant pores to generate additional electrical output. Reproduced with permission.^[^ ^87^ ^]^ Copyright 2021, Wiley‐VCH. d) A gel‐like triboelectric material made from carrots with a lot of mobile ions. Reproduced with permission.^[88]^ Copyright 2020, The American Association for the Advancement of Science. e) Triboelectric materials made of HEC have good transparency, excellent softness, and outstanding triboelectric properties. Reproduced with permission.^[^ ^89a^ ^]^ Copyright 2022, Elsevier. f) The positive and negative triboelectric materials of TENG are both composed of cellulose, which is crepe cellulose paper and nitrocellulose membrane, respectively. Reproduced with permission.^[^ ^89b^ ^]^ Copyright 2019, Elsevier. g) The modified CNF film with hydrophobicity. Reproduced with permission.^[^ ^90^ ^]^ Copyright 2020, American Chemical Society. h) The surface potential of CNFs is tailored by silane coupling agents. Reproduced with permission.^[^ ^79^ ^]^ Copyright 2021, Elsevier.

Paper is a common cellulosic triboelectric material.^[^ ^82^ ^]^ **Figure** **4**a shows a paper‐based TENG composed of simple repeating units folded together, which is simple to manufacture, cost‐effective, and practical.^[^ ^83^ ^]^ Wood plays an important role in architecture, engineering, and furniture.^[^ ^84^ ^]^ As shown in Figure 4b, Sun et al.^[^ ^85^ ^]^ functionalized the surface of native wood with a metal‐organic framework (MOF) and poly (dimethylsiloxane) (PDMS), respectively. And their effects on the surface roughness of wood‐based triboelectric materials were investigated. This approach greatly enhances the otherwise negligible triboelectric behavior of native wood. The reinforced wood‐based TENG fabricated on this basis can be used as self‐powered devices in future smart homes. Cellulosic aerogels are widely used as triboelectric materials because of their low density, high porosity, and large specific surface area.^[^ ^86^ ^]^ Cellulose II aerogel with abundant mesopores provides a larger surface area, effectively improving the contact efficiency, and its internal uniform continuous network structure leads to excellent mechanical response sensitivity and high electrical output (Figure 4c).^[^ ^87^ ^]^ Figure 4d shows a CNF film synthesized from a regenerated carrot combined with calcium chloride (CaCl_2_);^[^ ^88^ ^]^ the enhanced ionic conduction conferred by the plant hydrogel significantly weakens the influence of water molecules on the output properties of hydrogel triboelectric materials. The high chemical reactivity facilitates the production of various cellulose derivatives, such as hydroxyethylcellulose, porous nitrocellulose (Figure 4e,f); both are excellent candidates for triboelectric materials.^[^ ^89^ ^]^ Surface modification of triboelectric materials has always been a trending topic, by which researchers aim to increase the surface polarity and number of functional groups in cellulosic triboelectric materials, with a view to enhancing the output performance of TENGs. Nie et al.^[^ ^90^ ^]^ performed aminosilane modification on CNF films (Figure 4g). Numerous amino groups enhanced the positive polarity, generating many surface charges. The silane on the film surface is a hydrophobic barrier, and the mobile ions generated in the wet environment significantly improve the moisture resistance. Additionally, fluorine‐containing groups are capable of improving the electron‐withdrawing ability and reducing the surface free energy to prepare hydrophobic negative triboelectric materials.^[^ ^91^ ^]^ Their study provides insights into further expanding the range of triboelectric materials. Liu et al.^[^ ^79^ ^]^ investigated the effect of functional groups with different polarities on the surface charge density of CNFs, which was tailored by adjusting the number and density of functional groups on the CNF surface (Figure 4h). They proposed a relatively systematic and improved mechanism to describe the influence of chemically tailored surfaces on the CE, guiding for enhancing the triboelectric charge density of cellulosic materials through surface modification. Other related studies are summarized in **Table** **1**.

**Table 1:** Representative cellulosic triboelectric materials are prepared by conventional methods

Cellulose form	Preparation methods	Paired triboelectric materials	Electricity output	Potential applications	Refs.
Printer paper	Origami technique	PTFE	Open‐circuit voltage (*V*_OC_): 20 V Short‐circuit current (*I*_SC_): 2 µA Power density: 0.14 W m^‐2^	Paper‐based portable power sources and pressure sensors	[83]
CNF	Fabrication of CNF into fiberboard by chemical‐free cold pressing method	Fluorinated ethylene propylene (FEP)	*V*_OC_: 30 V *I*_SC_: 90 µA	Fiber‐based flooring for energy harvesting	[93]
Various commercial wrapping papers containing cellulose	Polydopamine molecular modification	Polyvinylidene fluoride (PVDF)	*V*_OC_: 1000 V *I*_SC_: 30 µA Transfer charge density: 76 µC m^−2^	Self‐powered anticorrosion and antifouling	[94]
Nitrocellulose	Finished film material	The user's finger	Output voltage over 60 V	Self‐triggered alarm system	[95]
CNF film	Chemical reaction approaches are employed to attach nitro groups and methyl groups to cellulose molecules	Nitro‐CNF paired with methyl‐CNF	*V*_OC_: 8 V *I*_SC_: 9 µA	–	[77]
Commercial printing paper	Tailoring	A porous PTFE thin film	*V*_OC_: 400 V *I*_SC_: 0.17 mA Power density: 53 W m^−2^	Paper‐based self‐powered electronic systems	[96]
Thin paper	Tailoring	FEP	*V*_OC_: 90 V Transfer charge (*Q*_SC_): 72 µC m^−2^	Sustainable power source for self‐powered electronics	[97]
Commercial paper cards	Tailoring	Teflon tape	*V*_OC_: 85 V *I*_SC_: 3.75 µA Power density: 39.8 µW cm^−2^	Inkjet printing, interface science for lab‐on‐chip biomedical micro/nanosystems	[98]
Ethylcellulose film	Inductively coupled plasma etching	Biocompatible medical 317L stainless steel	*V*_OC_: 245 V *I*_SC_: 50 µA	Biomedical science	[99]
Bacterial nanocellulose	Nitration method	Cu	Transfer charge density: 8.1 µC m^−2^ Power density: 4.8 mW m^−2^	Biomedical monitoring system	[35]
CNC	Deposition of CNC suspension droplets on ITO conductive films	FEP	*V*_OC_: 130 V *I*_SC_: 15 µA	Self‐powered handheld printer	[100]
CNF	High‐pressure homogenization of natural cellulose followed by vacuum filtration	Ag nanowires layer	*V*_OC_: 21 V *I*_SC_: 2.5 µA Power density: 693 mW m^−2^	Electronic paper	[101]
Cellulose paper	The two pieces of as‐prepared conductive paper were attached to pure tissue paper without overlap by using commercial glue.	Polyvinyl chloride (PVC) thin film	*V*_OC_ reaches 100V	Ambient energy harvesting and human motion detection	[102]
Cellulose acetate nanofibers	Electrostatic spinning	Polyethersulfone/carbon black/polystyrene (PES/C/PS) composite nanofibrous membrane	Power density: 0.13 W m^−2^	Wearable electronics and self‐powered systems	[103]
Crepe cellulose paper	‐	Nitrocellulose membrane	Power density: 16.1 W m^−2^	Paper piano for self‐powered human‐machine interfacing	[89b]
Natural balsa wood	Partial removal of lignin/hemicellulose from natural wood followed by hot pressing	PTFE	*V*_OC_: 81 V *I*_SC_: 1.8 µA *Q*_SC_: 36 µC m^−2^	Self‐powered sensing in athletic big data analytics	[15]
CNF	3D printing	PDMS	Power density: 29 W m^−2^	Self‐powered sensor	[104]
Natural New Zealand Pine	–	PTFE	*V*_OC_: 220 ± 20 V *I*_SC_: 5.8±0.5 µA Power density: 158.2 mW cm^−2^	Self‐triggered light switch, self‐triggered doorbell, self‐triggered floor	[105]
Natural wood	Modified with MOF materials and PDMS, respectively	Modified wood	*V*_OC_: 24.3 V *I*_SC_: 0.32 µA	Electrochromic windows	[85]
Carboxymethyl cellulose sodium	Electrostatic spinning	Carboxymethyl chitosan	*V*_OC_: 0.25 V *I*_SC_: 28 nA Power density: 120 mW cm^−2^	Baby care	[106]
Regenerated cellulose	Dissolving and regenerating natural cellulose	Cellophane film	*V*_OC_: 736 V *I*_SC_: 66.5 µA Power density: 307 W cm^−2^	High‐performance green TENGs for energy harvesting and self‐powered sensing	[81]
CNF	Perfluorosilane modification	Polyamide	*V*_OC_: 28.5 V Power density: 1.35 µW cm^−2^	Hydrophobicity‐enhanced energy harvester	[91]
Cellulose II aerogel	Natural cellulose is dissolved, regenerated, solvent exchanged, and freeze‐dried	PTFE	Power density: 127 mW cm^−2^	Human movement monitoring	[87]
Cellulose‐rich wheat straw	Cut the wheat straw into thin slices and stick them on the copper foil	FEP	*V*_OC_: 250 V *I*_SC_: 12 µA Power density: 404 mW cm^−2^	Wind velocity sensor	[107]
Hydroxyethyl cellulose film	Hydroxyethyl cellulose was fabricated from the reaction of alkali cellulose with ethylene oxide or ethylene chlorohydrin	PDMS	*V*_OC_: 584 V *I*_SC_: 41 µA *Q*_SC_: 96 nC	Omnidirectional wind energy harvester for self‐powered agro‐environmental information sensing	[89a]
Cotton	–	PTFE	*V*_OC_: 2500 V *I*_SC_: 85 µA *Q*_SC_: 1.1 µC	Smart agriculture	[108]
Cotton	–	FEP	*V*_OC_: 782 V *I*_SC_: 8.9 µA Power density: 1.89 mW	Wind and water energy harvester	[109]
Wood	The natural wood was chemically boiled in a mixed solution of NaOH and Na_2_SO_3_ and then hot‐pressed.	PTFE	Output voltage over 38V	Smart home control	[110]

Material‐structure and surface‐topography modifications increase only the contact area but not the inherent energy‐harvesting capacity of the material (unchanged), effectively resulting in a limited improvement. Surface functionalization is instrumental in enhancing the polarity of cellulosic triboelectric materials. However, the surface layer gradually wears out with increasing usage, which is not conducive to the long‐term usage of the TENG.^[^ ^92^ ^]^ To achieve a substantial and stable improvement in the cellulosic triboelectric properties, surface‐charge‐density enhancement via dielectric modulation should be focused on.

### 2.4 Potential for Dielectric Modulation of Cellulosic Triboelectric Materials

**Figure 5:**
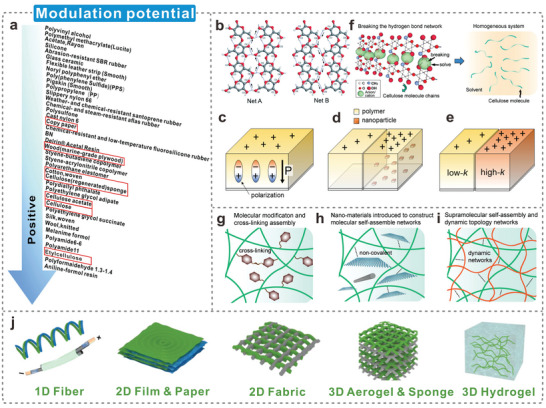
Potential and advantages of dielectric modulation of cellulose. a) Triboelectric series tending to positive polarity. Reproduced with permission.^[^ ^112^ ^]^ Copyright 2020, Wiley‐VCH. b) Two coexisting hydrogen bond networks in cellulose. Reproduced with permission.^[^ ^113^ ^]^ Copyright 2011, Royal Society of Chemistry. c–e) The polarization response of triboelectric materials is enhanced by adding nanofillers. Reproduced with permission.^[^ ^76^ ^]^ Copyright 2021, Wiley‐VCH. f) Solvent molecules disrupt the hydrogen bond network in cellulose molecules. Reproduced with permission.^[^ ^125^ ^]^ Copyright 2021, Wiley‐VCH. g–i) Molecular modification and chemical/physical cross‐linking assembly, nanomaterial hybridization, and supramolecular self‐assembly were used to prepare cellulosic functional materials, respectively. Reproduced with permission.^[^ ^125^ ^]^ Copyright 2021, Wiley‐VCH. j) Schematic diagram of the structure of the nanocellulose composite. Reproduced with permission.^[^ ^126^ ^]^ Copyright 2021, Wiley‐VCH.

The greater the difference in the electron‐withdrawing and electron‐donating capabilities of two triboelectric materials, the more triboelectric charges are generated during the contact.^[^ ^111^ ^]^ Several studies tested the tribopolarity of various materials and organized them from the most negative to positive tribopolarity to form the triboelectric series shown in **Figure** **5**a.^[^ ^112^ ^]^ Cellulose and its derived materials occupy a higher position in the triboelectric positive polarity series. The stronger electron‐donating ability prompts them to generate more triboelectric charges when paired with most materials. The coexisting hydrogen‐bonded networks of cellulose molecules enable excellent mechanical properties, higher thermal stability, flexibility, and tunable dielectric properties (Figure 5b).^[^ ^113^ ^]^ Greater polarization was achieved inside the cellulosic material via dielectric modulation (Figure 5c). Creating more interfacial regions in the cellulosic matrix by adding common fillers (Figure 5d) or high k fillers (Figure 5e) induces a stronger polarization response, which leads to higher surface charges.^[^ ^114^ ^]^ The type of matrix, reaction properties, molecular structure, and size and loading of the filler significantly affect the dielectric constant and dielectric loss of the composite.^[^ ^115^ ^]^ Thus, polymers for dielectric modulation must possess high chemical reactivity, stability, compatibility, assemblability, and wide‐range sizability for easy design. The hydrogen bond network of cellulose can be broken, and cellulose can be completely segregated into monomolecular chains rapidly using physical approaches (Figure 5f);^[^ ^116^ ^]^ this facilitates the molecular‐scale design of cellulosic materials. Many high‐performance tunable design strategies have been developed for cellulose, e.g., molecular modification, physical/chemical crosslinking assembly (Figure 5g),^[^ ^117^ ^]^ nanomaterial hybridization (Figure 5h),^[^ ^118^ ^]^ and supramolecular self‐assembly (Figure 5i).^[^ ^119^ ^]^ These methods provide a feasible strategy to fabricate cellulosic materials with novel and unique properties. Recently, cellulosic nanomaterials have attracted significant interest in composite materials research, and many advanced synthesis approaches and technologies have been proposed—surface modification (for compatibility enhancement), solution methods, melt methods, in situ polymerization, and layer‐by‐layer methods.^[^ ^120^ ^]^ Advanced preparation methods and technologies have provided more possibilities for the dielectric modulation of cellulose, enabling various types of fillers with different sizes to better combine with the cellulosic matrix and make them perform as expected. Moreover, continuous manufacturing and molding technologies have been developed–spinning (1D fibrous materials);^[^ ^121^ ^]^ continuous casting and spraying (2D films and fabrics);^[^ ^122^ ^]^ and extrusion, injection molding, and 3D printing (3D gels) (Figure 5j).^[^ ^123^ ^]^ Such cellulosic nanocomposites, with their multifunctionality, good compatibility, and inherent advantages, meet the requirements of cellulose‐based TENGs for various applications.^[^ ^124^ ^]^

As a natural positive triboelectric material, cellulose provides impetus to the long‐term development of TENGs owing to its excellent environmental friendliness and sustainability. High mechanical stability, processability, and assemblability, in combination with advanced material design strategies and fabrication processes, would enable cellulose to meet the requirements of dielectric modulation. This shows great promise for dielectric modulation and merits further discussion and exploration.

## 3 Dielectric Modulation Mechanisms of Cellulosic Triboelectric Materials

The theories for dielectric modulation of cellulosic triboelectric materials are discussed in this section. First, the effects of the dielectric properties on the TENG output are analyzed from the perspectives of dielectric constant and polarization. Subsequently, many theoretical models for predicting the effective dielectric constant of cellulosic materials are presented, comparing the emphasis between them. Additionally, the negative effects due to threshold phenomena during dielectric modulation of cellulosic triboelectric materials are analyzed from the perspective of percolation effects. Finally, some commonly used dielectric characterization methods are summarized here, and the technical characteristics between them are compared.

### 3.1 Dielectric Constant and Polarization Mechanism of Cellulosic Triboelectric Materials

**Figure 6:**
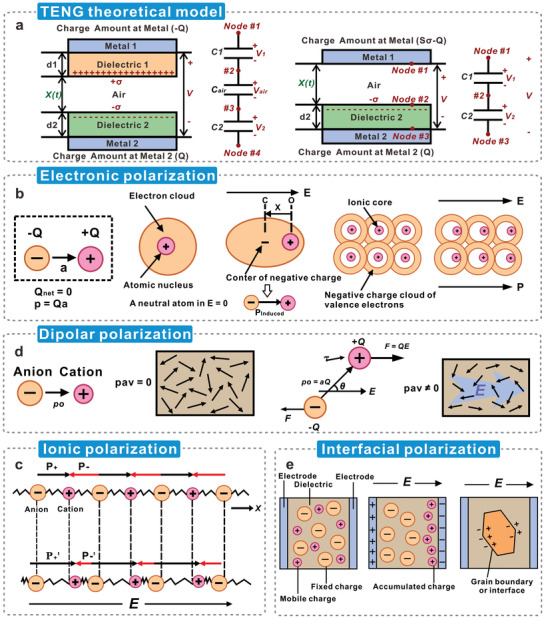
Dielectric constant and polarization mechanism of triboelectric materials. a) Theoretical models of dielectric‐to‐dielectric and conductor‐to‐dielectric TENGs. Reproduced with permission.^[^ ^127^ ^]^ Copyright 2015, Elsevier. b) Electrons or electron clouds in atoms are deflected by an electric field, causing electron polarization, c) Ionic polarization arises from the relative displacement that occurs between oppositely charged ions in an ionic crystal, d) Under the action of an external electric field, the dipoles in the material are oriented and aligned, causing dipole polarization, and e) Interfacial polarization occurs at the interface of heterogeneous materials due to their differences in polarity and conductivity. Reproduced with permission.^[^ ^141^ ^]^ Copyright 2019, Elsevier.

For a typical CS‐TENG (**Figure** **6**a),^[^ ^127^ ^]^ its electrical properties are described by the relationship between the induced voltage (V), amount of transferred charges (*Q*), and displacement of the triboelectric layers (*x*).^[^ ^128^ ^]^

The *V–Q–x* relationship can be unified as Equation 1

(1) $$V=-\frac{Q}{S\varepsilon_{0}}\left(d_{0}+x\left(t\right)\right)+\frac{\sigma x(t)}{\varepsilon_{0}}$$

The open‐circuit voltage (*V*_OC_) is given by Equation 2

(2) $$V_{\mathrm{OC}}=\frac{\sigma x(t)}{\varepsilon_{0}}$$

In the short‐circuit (SC) state, *V* is 0. The transferred charge *Q*_SC_ is given by Equation 3

(3) $$Q_{\mathrm{SC}}=\frac{S\sigma x(t)}{d_{0}+x\left(t\right)}$$

The short‐circuit current (*I*_SC_) is given by Equation 4

(4) $$I_{\mathrm{SC}}=\frac{dQ_{\mathrm{SC}}}{dt}=\frac{S\sigma d_{0}}{{\left(d_{0}+x\left(t\right)\right)}^{2}}\frac{dx}{dt}=\frac{S\sigma d_{0}v\left(t\right)}{{\left(d_{0}+x\left(t\right)\right)}^{2}}$$

Therefore, the output performance of TENG directly depends on the surface charge density (*σ*) and effective dielectric thickness (*d_0_*) of the triboelectric materials. Additionally, in the parallel plate capacitor model, *σ* is related to the capacitance (*C*) of the dielectric layer, the relationship can be represented in Equations 5 and 6:^[^ ^129^ ^]^

(5) $$\sigma =\frac{CV}{S}$$

(6) $$C=\frac{S\varepsilon_{r}\varepsilon_{0}}{d}$$

A larger dielectric constant and thinner size are the keys to improving the output performance of TENG. Recent studies have shown that increasing the dielectric constant can effectively increase the total transferred charge density (*σ*′).^[^ ^130^ ^]^ Its expression is given by Equation 7

(7) $$\sigma^{\prime }=\frac{-\sigma_{0}d_{\mathrm{gap}}}{d_{\mathrm{gap}}+d_{0}/\varepsilon_{r}}$$

where *σ*_0_ is the triboelectric charge density at the equilibrium state, *d*_gap_ and *d*_0_ are the gap distance and thickness of the dielectric film, respectively, and *ε*_r_ is the dielectric constant. Increasing the dielectric constant of triboelectric materials can effectively improve the output performance of TENG at a fixed surface charge density.^[^ ^130^ ^]^ Moreover, an increase in the dielectric constant is beneficial for increasing the surface charge accumulated during the contact.^[^ ^131^ ^]^ Owing to this dual effect, an increase in the dielectric constant significantly improves the triboelectric properties.

Dielectric constant (*ε_r_*) is defined as the ability of a material to polarize in an alternating current electric field. Its expression is as follows (Equation 8):^[^ ^132^ ^]^

(8) $$\varepsilon_{r}=\varepsilon_{r}^{\prime }-i\cdot \varepsilon_{r}^{\prime \prime }$$

The real part of the dielectric constant (*ε*_r_′) represents the polarization response of the dielectric to an applied electric field and is related to the capacitance of the material. The imaginary part of the dielectric constant (*ε*″_r_) represents energy dissipation, such as heat or other forms of energy loss, caused by the inability of the material to produce real‐time polarization with changes in the oscillation frequency.^[^ ^133^ ^]^

The dielectric properties of cellulosic triboelectric materials are closely related to the polarization mechanism. Polarization is defined as the sum of the total dipole moments in unit volume of the dielectric and is related to the dielectric constant in a uniform electric field, as shown in Equation 9:^[^ ^134^ ^]^

(9) $$P=\left(\varepsilon_{r}-1\right)\varepsilon_{o}E$$

where *P* represents the dielectric polarization, *E* is the applied electric field, and *ε*_0_ is the dielectric constant of vacuum. In general, dielectric polarization can be divided into four types: electronic polarization (*P*_e_), ionic polarization (*P*_i_), dipolar polarization (*P*_d_), and interfacial polarization (*P*_int_).^[^ ^135^ ^]^ According to the mechanism of charge movement, the above polarizations can be roughly divided into three categories: distortion polarization caused by the distortion of the electron cloud by the external field, displacement polarization caused by the displacement of the free charge, and steering polarization caused by the rearrangement of the dipole under the action of the external field.^[^ ^136^ ^]^ *P*_e_ originates from the induced dipole moment due to the displacement of electrons or electron clouds in atoms under the action of *E* (Figure 6b),^[^ ^137^ ^]^ which belongs to distortion polarization. However, the increase in the dielectric constant caused by *P*_e_ is often limited by restraints on the intrinsic bonding structure of the cellulose molecules. Under the action of *E*, a relative displacement occurs between oppositely charged ions in the ionic crystal, leading to *P_i_* (Figure 6c).^[^ ^138^ ^]^ This polarization belongs to the displacement polarization of a single‐phase dielectric. The short‐range displacement of bound charges and the long‐range displacement of free charges can lead to *P*_i_.^[^ ^136^, ^139^ ^]^ In cellulosic triboelectric materials, certain ionic components such as NaCl and LiCl can increase the surface charge density via *P*_i_.^[^ ^140^ ^]^ Additionally, the structure and physicochemical properties of cellulosic triboelectric materials change because of the decrease in ionic bond components and increase in covalent bond components caused by *P*_i_. *P*_d_ is also known as orientational polarization or steering polarization, which stems from the intrinsic orientation of polar molecules or the induced orientation caused by the asymmetric deformation of nuclei in nonpolar molecules (Figure 6d).^[^ ^136^ ^]^ When *E* is applied, the dipoles tend to align parallel to the direction of *E*; accordingly, the material exhibits a net polarization.^[^ ^141^ ^]^ Polymers can be classified into polar and nonpolar polymers based on their mean dipole moments.^[^ ^142^ ^]^ Their polar properties depend on the presence and location of polar groups as well as the chain geometry. The underlying reason may be the difference in electronegativity between bond elements. The more electronegative element, or more abundant element, pulls the electron cloud to its side, causing the positive and negative charges to separate.^[^ ^135^ ^]^ The naturally weaved chemical structure and the spatial asymmetry of the molecules endow cellulose with strong polarity.^[^ ^143^ ^]^ Furthermore, cellulose chains exhibit significant dipole moments along the length of the chain.^[^ ^144^ ^]^ The surface of cellulose contains a large number of polar hydroxyl groups and the dipole polarization is generated by the applied electric field, so the dielectric constant of cellulose is higher than that of ordinary polymers, usually ranging from 3.9 to 7.5.^[^ ^145^ ^]^ It is exciting that cellulosic nanomaterials exhibit highly promising dipolar properties. Due to the structural anisotropy of the cellulose nanocrystals, the dipole moment in the direction parallel to the rod main axis is much stronger than the one in the perpendicular direction.^[^ ^146^ ^]^ This shows promising results for the construction of cellulosic materials with high intrinsic dielectric properties. *P*_int_, also known as Maxwell‐Wagner‐Sillars polarization, occurs at the interface of different media.^[^ ^147^ ^]^ The dielectrics on both sides of the interface have volume effects and charge accumulation effects owing to different polarities or conductive properties,^[^ ^148^ ^]^ and electrons or ions in the dielectrics accumulate at the interface between the two regions in the presence of *E* (Figure 6e).^[^ ^149^ ^]^ Therefore it can be considered as displacement polarization in multiphase dielectrics.^[^ ^150^ ^]^ In cellulosic triboelectric materials, various factors such as impurities or defects inside the material, crystal–amorphous interfaces, and interfaces between films and electrodes could lead to *P*_int_.^[^ ^151^ ^]^ Among them, generating a strong Pint by doping fillers is the most facile and effective modulation method, wherein the degree of polarization can be regulated by adjusting the filler loading. In the research progress in recent years, doping functional fillers to build strong interfacial polarization is the most commonly used method, and also achieved good results. The remainder of this review will focus on issues and control points related to interface polarization.

### 3.2 Interface Polarization Effects in Dielectric Modulation of Cellulosic Triboelectric Materials

The crystalline and amorphous regions usually undergo some *P*_int_ at the interface owing to the difference in the electron cloud densities,^[^ ^152^ ^]^ which is an inherent dielectric property of cellulosic triboelectric materials. The loading of fillers generates many interfacial regions inside the cellulosic material, and the huge difference in electrical properties of the filler and cellulosic material further intensifies *P*_int_.^[^ ^153^ ^]^ To better understand the specific process, two classic models are introduced.

**Figure 7:**
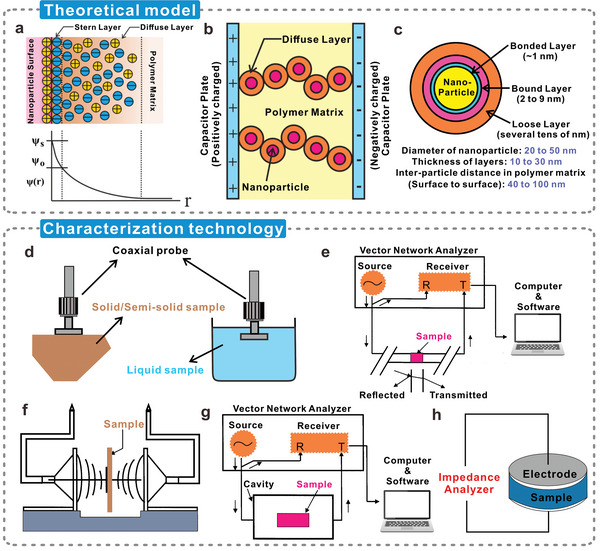
Theoretical models on dielectric modulation and characterization techniques for dielectric constant. a) The diffuse electric double layer in Lewis's model, and the resulting potential distribution *ψ*(r), b) conduction through diffusive bilayers in composite systems, and c) a multinucleus model of the nanoparticle‐polymer interface. Reproduced with permission.^[^ ^135^ ^]^ Copyright 2017, American Chemical Society. d) Coaxial probe method for measuring the dielectric properties of solid and liquid samples, e) equipment diagram and flowchart for the transmission line method, f) free space method for measuring large flat solid samples, g) the sample to be tested is placed in the resonant cavity used by the resonant cavity method, and h) the sample is sandwiched between two parallel electrode plates to form a capacitor for dielectric measurements using the capacitive plate method. Reproduced with permission.^[^ ^194^ ^]^ Copyright 2012, Information Technology and Electrical Engineering Publishers.

The first is Lewis’ model,^[^ ^154^ ^]^ which applies to nanofillers with random shapes. The addition of nanofillers to cellulosic materials results in the formation of numerous interfaces with nanometric dimensions, depending on the size and dispersion of the nanofillers (**Figure** **7**a).^[^ ^155^ ^]^ The interface is usually divided into three layers. Close to the nanoparticle, a charged layer will be formed related to its surface state, arising from immobile charged impurities, trapped carriers, and mobile electrons and holes.^[^ ^156^ ^]^ The strength and extent of this layer will depend on whether the nanofiller is intrinsically insulating or conductive. Meanwhile, the cellulosic matrix responds by generating equal opposite charges around the nanofillers, forming a nanometer‐sized Stern (Helmholtz) layer at the interface, which contains small molecules, special absorbed ions, and solvated ions, and cannot move freely.^[^ ^157^ ^]^ The outer Helmholtz plane (OHP), on the side of the Stern layer away from the nanoparticle, is determined by the nearest approach of ions, which, attracted by the excess charge on the nanoparticle, have drifted to the surface.^[^ ^156^ ^]^ Typically, there is a diffused and decreasing Gouy‐Chapman space charge layer outside the OHP, whose range depends on the concentration of charged ions in the dielectric.^[^ ^154^ ^]^ If the polymer matrix is highly conductive, the layer will shrink into the OHP, but in an insulating material like cellulose, it may extend to 10 nm or more.^[^ ^156^ ^]^ Some ions can be transferred and diffused in the Gouy‐Chapman space charge layer, and their sources may be the external environment,^[^ ^158^ ^]^ and additives, which are used to improve the interfacial interaction between nanoparticles and cellulose.^[^ ^120^, ^159^ ^]^ Functional additives, such as surfactants,^[^ ^160^ ^]^ crosslinkers,^[^ ^161^ ^]^ plasticizers,^[^ ^162^ ^]^ and ionic liquids (for specific materials),^[^ ^163^ ^]^ are typically added at the composite stage to enhance the stability and processing properties of cellulosic nanocomposites, providing a large number and multiple types of diffusing ions for the nanocomposites in the meantime.

The charged nanoparticles can cause charge redistribution in the matrix under the action of Coulomb force, forming a diffuse electric double layer composed of a Stern layer and a Gouy‐Chapman layer, which is collectively described as the Stern/Gouy‐Chapman double layer model.^[^ ^164^ ^]^ This region of mobile charge plays a significant role in determining the dielectric properties of cellulosic nanocomposites, mainly affecting the percolation threshold of fillers. The conduction process of the nanofillers through the diffused electric double layer is shown in Figure 7b.^[^ ^135^ ^]^ It is important to note that the ideal Lewis model is more suitable for elaborating the interface state between nanofillers with smooth surfaces and dielectric matrix.^[^ ^165^ ^]^ However, in reality, defects and surface roughness on the surface of nanoparticles inevitably exist, affecting the resistance and charge distribution and impeding the interface polarization response.^[^ ^166^ ^]^ To overcome this issue, some modification methods can be used for improvement, such as surface modification and coating of nanofillers.^[^ ^167^ ^]^

Figure 7a presents the potential distribution, *Ψ*(*r*), with the distance from the nanoparticle surface.^[^ ^135^ ^]^ The Gouy‐Chapman equation (Equation 10) shows the potential change in the diffusion part of the double layer, starting from the Stern layer:^[^ ^168^ ^]^

(10) $$\tanh\left(\frac{z_{i}e\psi \left(r\right)}{4kT}\right)=\tanh\left(\frac{z_{i}e\psi \left(o\right)}{4kT}\right)e^{-Kr}$$

where *Ψ*(*r*) is the potential distribution function that varies with distance, *k* is the Boltzmann constant, *K* is the Debye‐Hückel parameter, and *z_i_* is the valence state of ionic species *i* in the bulk matrix.

The second is Tanaka's model (Figure 7c),^[^ ^169^ ^]^ which is suitable for describing interfaces formed by physical, chemical, or electrical means because of the spherical inorganic nanofillers added to a cellulosic matrix. This model is often used to describe the interaction between polymers such as cellulose and spherical inorganic nanoparticles.^[^ ^170^ ^]^ When spherical nanoparticles are added to the polymer matrix of cellulose, the interface of the nanoparticles can be divided into three layers—bonded layer, bound layer, and loose layer—from the center of the particle to the exterior.^[^ ^171^ ^]^ The bonded layer corresponds to the transition layer, wherein the inorganic and organic layers are tightly bound by chemical bonds through the coupling agents. Strong bonding is associated with hydrogen bonds, van der Waals forces, ionic bonds, and covalent bonds.^[^ ^172^ ^]^ When dispersing inorganic nanofillers into a polymer matrix, such as cellulose, it is important to consider the affinity between their contacting surfaces. To achieve a good interface, coupling agents are commonly used in polymer‐inorganic composites to reduce the difference in surface tension between the two materials.^[^ ^169^ ^]^ One common practice is to use silanes for coupling, which are used to couple organic polymers with inorganic filler particles.^[^ ^173^ ^]^ Ethoxysilane,^[^ ^174^ ^]^ methoxysilane,^[^ ^175^ ^]^ and aminosilane^[^ ^176^ ^]^ are among the most commonly used silanes. These surface treatments are used for inorganic nanofillers to create chemical bonds, for example, Si—O or Si—N bonds, between organic and inorganic materials.^[^ ^177^ ^]^ The mechanism of action is mainly through the reaction of silicon bonds with surface chemical groups, including the participation of silicon radical or methyl silicon radical.^[^ ^178^ ^]^ The bound layer is an interfacial region whose thickness depends on the interaction between cellulose and nanoparticles. The polymer chains of cellulose in the bound layer form structures around the filler nanoparticle that affect chain folding, mobility, and conformation.^[^ ^170^ ^]^ The loose layer is a region that is loosely coupled; it interacts with the bound layer. The polymer matrix in this region has different chain conformations, mobilities, free volumes, and crystallinities.^[^ ^179^ ^]^ This layer is attributed to the contribution of the reduction in the free volume of the composite. On the one hand, the dipole orientation of polar radicals in the bound layer is adversely affected by the binding effect. On the other hand, the loose layers reduce the free volume. Both these effects synergistically decrease the dielectric constant. Tanaka's model investigates various possibilities for tailoring the dielectric properties of polymer nanocomposites, thereby providing insights into cellulosic nanocomposite triboelectric materials.^[^ ^180^ ^]^

### 3.3 Prediction Models for Effective Dielectric Constant of Cellulosic Triboelectric Materials

The effective dielectric constant of cellulosic nanocomposite triboelectric materials is affected by many factors, including the individual dielectric constants of the fillers and cellulosic matrix, filler loadings, and interactions among them. Empirical formulae and theoretical equations have been proposed to predict the dielectric constants of nanocomposites under different conditions, which are also applicable to cellulosic triboelectric materials. The most representative ones—Lichtenecker formula and Maxwell‐Garnett equations—are presented here as examples, and other models and formulae are listed in **Table** **2**.

**Table 2:** Various mathematical models for calculating the effective dielectric constant of cellulosic nanocomposite triboelectric materials

Models	Formula	Important points	Refs.
Maxwell−Garnett equation	$\varepsilon_{\mathrm{eff}}=\varepsilon_{m}\left[1+\frac{3\varphi_{f}\left(\varepsilon_{f}-\varepsilon_{m}\right)}{\varphi_{m}\left(\varepsilon_{f}-\varepsilon_{m}\right)+3\varepsilon_{m}}\right]$	– It is suitable for low loadings of spherical nanofillers	[184, 186]
	$\varepsilon_{\mathrm{eff}}=\varepsilon_{m}+\varepsilon_{m}\frac{\frac{\varphi_{f}}{3}\sum_{j=x,y,z}\frac{\varepsilon_{f}-\varepsilon_{m}}{\varepsilon_{m}+A_{j}\left(\varepsilon_{f}-\varepsilon_{m}\right)}}{1-\frac{\varphi_{f}}{3}\sum_{j=x,y,z}\frac{A_{j}\left(\varepsilon_{f}-\varepsilon_{m}\right)}{\varepsilon_{m}+A_{j}\left(\varepsilon_{f}-\varepsilon_{m}\right)}}$	– For ellipsoidal nanofillers	[187]
Wiener bounds	*ε*_eff_ = *φ*_f_ *ε*_f_ + *φ*_m_ *ε*_m_(parallel)	– The maximum or minimum value of the effective dielectric constant is given:*φ*_f_ + *φ*_m_ = 1	[184]
	$\frac{1}{\varepsilon_{\mathrm{eff}}}=\frac{\varphi_{f}}{\varepsilon_{f}}+\frac{\varphi_{m}}{\varepsilon_{m}}\left(\mathrm{series}\right)$		
Lichtenecker logarithmic law	$\varepsilon_{\mathrm{eff}}^{\alpha }=\varphi_{f}\varepsilon_{f}^{\alpha }+\varphi_{m}\varepsilon_{m}^{\alpha }$	– Indicating anisotropy,− 1 ≤ *α* ≤ 1	[182]
Modified Rother−Lichtenecker model	ln *ε*_eff_ = ln *ε*_m_ + *φ*(1 − *η*)ln (*ε*_f_/*ε*_m_)	– Includes shape‐related parameters	[188]
Sillars or Landzu−Lifshitz rules	$\varepsilon_{\mathrm{eff}}=\varepsilon_{m}\left[1+\frac{3\varphi_{f}\left(\varepsilon_{f}-\varepsilon_{m}\right)}{2\varepsilon_{m}+\varepsilon_{f}}\right]$	– Limited to filler and matrix conductivity, only suitable for lower filler loads	[189]
Yamada model	$\varepsilon_{\mathrm{eff}}=\varepsilon_{m}\left[1+\frac{\eta \varphi_{f}\left(\varepsilon_{f}-\varepsilon_{m}\right)}{\eta \varepsilon_{m}+\left(1-\varphi_{f}\right)\left(\varepsilon_{f}-\varepsilon_{m}\right)}\right]$	– It is assumed that the filler is uniformly distributed in the matrix	[190]
Bruggeman self‐consistent effective medium approximation	$\frac{\varepsilon_{f}-\varepsilon_{\mathrm{eff}}}{\varepsilon_{\mathrm{eff}}^{1/3}}=\frac{\left(1-\varphi_{f}\right)\left(\varepsilon_{f}-\varepsilon_{m}\right)}{\varepsilon_{m}^{1/3}}$	– Good for higher filler content, but doesn't give accurate results near threshold	[184]
Jaysundere−Smith equation	$\varepsilon_{\mathrm{eff}}=\frac{\varepsilon_{m}\varphi_{m}+\varepsilon_{f}\varphi_{f}\frac{3\varepsilon_{m}}{2\varepsilon_{m}+\varepsilon_{f}}\left[1+3\varphi_{f}\frac{\varepsilon_{f}-\varepsilon_{m}}{2\varepsilon_{m}+\varepsilon_{f}}\right]}{\varphi_{m}+\varphi_{f}\frac{3\varepsilon_{m}}{2\varepsilon_{m}+\varepsilon_{f}}\left[1+3\varphi_{f}\frac{\varepsilon_{f}-\varepsilon_{m}}{2\varepsilon_{m}+\varepsilon_{f}}\right]}$	– Considering the interaction between fillers	[191]
Effective medium percolation theory (EMPT) mode	$\varepsilon_{\mathrm{eff}}=\varepsilon_{m}\left[1+\frac{\varphi_{f}\left(\varepsilon_{f}-\varepsilon_{m}\right)}{\varepsilon_{f}+\eta \left(1-\varphi_{f}\right)\left(\varepsilon_{f}-\varepsilon_{m}\right)}\right]{|\frac{\varphi_{c}-\varphi_{p}}{\varphi_{p}}|}^{-q}$	– Includes EMT and percolation theory	[192]

Lichtenecker formula is the most commonly used method for calculating the effective dielectric constant of polymer nanocomposites.^[^ ^181^ ^]^ It is a logarithmic mixing formula suitable for heterogeneous media mixed with multiphase materials, expressed as Equation 11:^[^ ^182^ ^]^

(11) $$\varepsilon_{\mathrm{eff}}^{\alpha }=\varphi_{f}\varepsilon_{f}^{a}+\varphi_{m}\varepsilon_{m}^{\alpha }$$

where *ε*_eff_ is the effective dielectric constant of the nanocomposite; *ε*_m_ and *ε*_f_ are the dielectric constants of the polymer matrix and nanofillers, respectively; and *φ*_f_ and *φ*_m_ are the volume fractions of the filler and matrix, respectively. Here, *α* varies from ‐1 to 1. Thus, this equation sets the upper and lower limits of the dielectric constant of a composite material.

Maxwell‐Garnett equation, also known as Maxwell‐Wagner equation, applies to low spherical filler loading.^[^ ^183^ ^]^ The equation has distinct linear properties, is relatively simple and easy to model, and is not limited by the resistivity of the filler or matrix in the polymer nanocomposites. The general expression is shown in Equation 12:^[^ ^184^ ^]^

(12) $$\varepsilon_{\mathrm{eff}}=\varepsilon_{m}\left[1+\frac{3\varphi_{f}\left(\varepsilon_{f}-\varepsilon_{m}\right)}{\varphi_{m}\left(\varepsilon_{f}-\varepsilon_{m}\right)+3\varepsilon_{m}}\right]$$

When the fillers are not standard spheroids but randomly oriented ellipsoids, the general formula is modified by a depolarization factor related to the deviation from sphericity, and the effective dielectric constant is given by Equation 13:^[^ ^185^ ^]^

(13) $$\varepsilon_{\mathrm{eff}}=\varepsilon_{m}+\varepsilon_{m}\frac{\frac{\varphi_{f}}{3}\sum_{j=x,y,z}\frac{\varepsilon_{f}-\varepsilon_{m}}{\varepsilon_{m}+A_{j}\left(\varepsilon_{f}-\varepsilon_{m}\right)}}{1-\frac{\varphi_{f}}{3}\sum_{j=x,y,z}\frac{A_{j}\left(\varepsilon_{f}-\varepsilon_{m}\right)}{\varepsilon_{m}+A_{j}\left(\varepsilon_{f}-\varepsilon_{m}\right)}}$$

where *A* is the depolarization factor. When *A* = 1/3, that is, it refers to spherical inclusions.

### 3.4 Characterization Techniques for Measuring the Dielectric Constant of Cellulosic Triboelectric Materials

The dielectric constant of a material is closely related to frequency.^[^ ^193^ ^]^ For dielectric measurements, however, no single technique can characterize all materials over the entire frequency band. Performing accurate dielectric measurements on different types of dielectric materials is very challenging. Because when characterizing materials, dielectric measurement depends on many factors, including frequency, temperature, required accuracy, properties and appearance of the sample, whether to contact the sample, whether to destroy the sample and the cost of the measurement.^[^ ^194^ ^]^ To better realize the dielectric modulation of cellulosic triboelectric materials and guide related research, some of the most popular and important dielectric measurement methods are briefly discussed here.

The coaxial probe method is one of the most convenient and commonly used techniques for measuring high frequencies (i.e., radio frequency and microwave).^[^ ^195^ ^]^ It is also known as coaxial probe or coaxial‐line probe or an open ended coaxial‐line method.^[^ ^196^ ^]^ A metallic probe on the coaxial line is used to sense the phase and magnitude of the reflected signal from the measurement device (Figure 7d). The tip of the probe is usually flat and uncurved to make contact with the sample. Therefore, it has quite high requirements on the surface flatness of the sample. For liquid samples, metal probes can be conveniently dipped directly into the sample. This method can perform measurements over a wide frequency range from 0.5 to 110 GHz,^[^ ^197^ ^]^ but it shows a bias for measurements on materials with low dielectric constants.^[^ ^198^ ^]^ Another method is to place the material sample in the center of a closed transmission line (Figure 7e),^[^ ^199^ ^]^ thus called the transmission line method. It has higher accuracy and sensitivity than the coaxial method, but its measurable frequency range is not as good as the coaxial method.^[^ ^194^, ^198^ ^]^ During the measurement, the dielectric constant of the material is theoretically calculated by using the reflection coefficient and the transmission coefficient as intermediate values.^[^ ^200^ ^]^ In terms of sample requirements, the sample to be measured must cover the entire cross‐section of a transmission line, generally in a circular geometry.^[^ ^199^ ^]^ Therefore, sample preparation for this method is relatively difficult and time‐consuming.

Both of the above two methods require the sample to be in contact with the measuring equipment, which inevitably contaminates or destroys the sample. The free space method is a typical non‐contact and nondestructive measurement technique.^[^ ^201^ ^]^ In this method, a solid sample with a large area, a flat surface, and uniform surfaces on both sides is placed in two horn antennas (Figure 7f).^[^ ^202^ ^]^ A solid sample combined with a vector network analyzer directs energy to or through the horn antennas.^[^ ^203^ ^]^ The advantage of this method is that the reflectance coefficient and transmittance coefficient can be obtained in a non‐contact manner. This is very beneficial for thin and flat materials.^[^ ^204^ ^]^ The resonant cavity method is also a common dielectric measurement technique that does not destroy the sample excessively (Figure 7g).^[^ ^199^ ^]^ It is suitable for homogeneous materials, has high accuracy, and is capable of measuring at high temperatures.^[^ ^198^ ^]^ The sample to be measured inserted in the tuning cavity will cause the shift of the resonant frequency. The dielectric constant of the material is calculated from the changes in the resonant frequency and quality factor in the tuned cavity.^[^ ^205^ ^]^ Typical measurement ranges for this method are from 50 MHz to over 100 GHz.^[^ ^206^ ^]^ Before measurement, each cavity needs to be calibrated to speed up the measurement of large batches of samples.^[^ ^207^ ^]^ Compared with other techniques, the sample preparation of this method is simple and fast, and it can work in a wide temperature range (140 °C to −20 °C).^[^ ^194^, ^198^ ^]^ There is the parallel electrode method, also known as the parallel plate capacitor method. A capacitor is formed by sandwiching a thin sheet of dielectric material between two parallel electrode plates (Figure 7h).^[^ ^208^ ^]^ Calculate the dielectric constant by the capacitance value. It has high accuracy and relatively low equipment requirements, and can be directly measured by a commercial LCR meter or impedance analyzer.^[^ ^209^ ^]^ Typically the frequency range is 20 Hz to 1 GHz.^[^ ^194^ ^]^ This method is very simple in sample preparation and equipment operation, and low in measurement cost, which is very suitable for small‐scale experiments in the laboratory.

The comparison of the above five methods is given in **Table** **3**. The resonant method is certainly preferable at lower and medium frequencies. Coaxial, resonant cavity, and free space methods are better suited for high frequencies. And before choosing an appropriate measurement method, the characteristics of the sample itself must be considered. A good characterization technique must provide high precision, low cost, simple and fast measurements of the sample material.

**Table 3:** A comparison of various dielectric measurement characterization techniques

Method	Advantages	Defects	Refs.
Coaxial probe	– Wide frequency range – Best for semisolids or liquids – Simple sample preparation – Isotropic and homogeneous material – High accuracy	– Calibration needs to be repeated – Air gaps can lead to errors	[199]
Transmission line	– High frequency – Suitable for both solid and liquid samples – Suitable for heterogeneous materials	– Complicated sample preparation – Cannot be used at low frequency	[194]
Free space	– Noncontacting – Wide frequency range – Easy sample preparation – No damage to the sample	– Diffraction effects at material edges affect measurement results – Complex calculation	[203]
Resonant cavity	– Suitable for both solid and liquid samples – High‐temperature measurement support – Highest accuracy	– Very small sample size	[199]
Parallel plate	– Higher accuracy – Simple device operation – Low cost of measurement	– Electrode polarization effect	[208, 210]

### 3.5 Threshold Phenomenon in Dielectric Modulation of Cellulosic Triboelectric Materials

**Figure 8:**
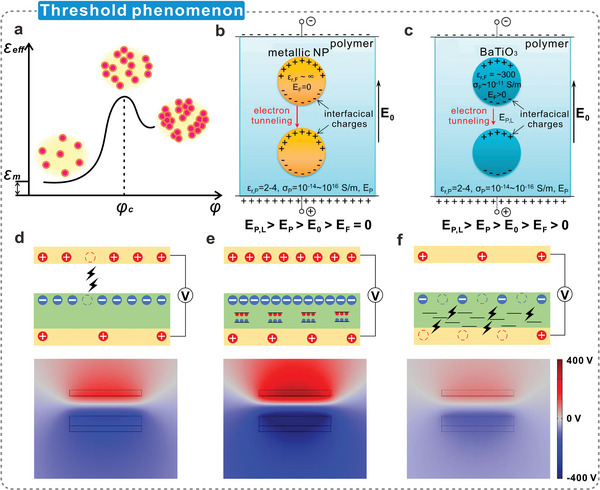
Threshold phenomenon in dielectric modulation of cellulosic triboelectric materials. a) Relationship between effective dielectric constant and filler volume fraction. Reproduced with permission.^[^ ^141^ ^]^ Copyright 2019, Elsevier. b) Interfacial polarization of metal nanoparticles in a dielectric polymer matrix, and c) interfacial polarization of ceramic nanoparticles in a dielectric polymer matrix. Reproduced with permission.^[^ ^215^ ^]^ Copyright 2014, American Chemical Society. d) The surface charge density of dielectric films is mainly limited by the air breakdown limit of TENG, e) the addition of fillers creates a large number of microcapacitors inside the triboelectric material, greatly increasing the surface charge density, and f) electrical breakdown between the triboelectric material and the back electrode will result in a reduction in the total surface charge. Reproduced with permission.^[219]^ Copyright 2021, The American Association for the Advancement of Science.

In the dielectric modulation of cellulosic triboelectric materials, the increase of the dielectric constant is not unlimited. During the doping of fillers to promote strong interfacial polarization, due to the inevitable voids, aggregation of fillers, and formation of conductive networks, an increase in dielectric loss usually results in a decrease in the charge‐trapping capability of triboelectric materials.^[^ ^211^ ^]^ At the same time, the close arrangement of fillers will reduce the effective contact area of the material, which will also adversely affect the contact efficiency of the TENG.^[^ ^212^ ^]^ The relationship between *ε*_eff_ and filler volume fraction (*φ*) of the polymer nanocomposites is shown in **Figure** **8**a.^[^ ^141^ ^]^ When *φ* in the cellulosic material reaches a critical value (i.e., the percolation threshold, *φ*_c_), the agglomerated fillers form a continuous conductive pathway and extend inside the material,^[^ ^213^ ^]^ resulting in a partial insulator‐to‐metal transition. In addition, the dielectric properties of the material change significantly, such as a low dielectric constant, high dielectric losses, and premature dielectric breakdown.^[^ ^214^ ^]^ The percolation threshold varies with the nanofiller type, geometric characteristics, and strength and extent of interparticle attractive interactions. Metal (Figure 8b) and ceramic (Figure 8c) nanoparticles are used as examples. Generally, ceramic nanoparticles have an extremely high dielectric constant,^[^ ^215^ ^]^ whereas the dielectric constant of metal nanoparticles can be considered to approach infinity. The significant difference in the dielectric constant of the inorganic nanoparticles and cellulosic matrix results in a nonuniform electric field distribution in the cellulosic nanocomposite dielectric.^[^ ^216^ ^]^ Because the electrical conductivity of both metal and ceramic nanofillers is higher than that of cellulosic materials, the free electrons in the metal and ceramic nanofillers are polarized and induce large‐area interfacial polarization when an external electric field is applied. These surface charges due to interfacial polarization are analogous to the surface plasmons in metal nanoparticles and the surface charges of insulating particles in electrorheological fluids.^[^ ^217^ ^]^ When two closely spaced polarized nanoparticles are aligned exactly in the field direction, the localized field in the polymer matrix between them increases further. The material interior generates electron tunneling or conduction, decreasing the dielectric constant and breakdown strength.

When the volume fraction of the filler reaches approximately *φ*_c_, the dielectric constant of the material increases drastically, which can be explained by the microcapacitor network.^[^ ^218^ ^]^ Inside the composite, a thin layer of dielectric sandwiched between conductive fillers can be considered a miniature capacitor. With a significant increase in the local electric field strength, each microcapacitor contributes to a larger capacitance as the volume fraction of the filler approaches *φ*_c_. This promotes the migration and accumulation of charge carriers at the interface, which increase with *φ* until the filler loading is very close to the threshold *φ_c_*. At this time, the distance between the filler particles is smaller than the range of electron tunneling or seepage that occurs upon direct contact. To further understand the specific performance of dielectric and percolation effects in TENGs, the corresponding potential distributions on dielectric layers and electrodes were simulated using finite element analysis (Figure 8d–f).^[^ ^219^ ^]^ According to Paschen's law,^[^ ^220^ ^]^ the maximum surface charge density of the material is limited by the air breakdown limit. Paschen's law describes the empirical relationship between the gaseous breakdown voltage (*V*_b_) and the product of the gas pressure (*P*) and gap distance (*d*), and is given by Equation 14:^[^ ^221^ ^]^

(14) $$V_{b}=\frac{APd}{\ln(Pd)+B}$$

where *A* and *B* are the constants determined by the composition and the pressure of the gas. For air at standard atmospheric pressure (atm, i.e., the conventional operation condition of a TENG), *A*  =  2.87 × 10^5^ V atm^−1^ m^−1^, and *B*  =  12.6.

Taking SE‐TENG in Figure 8d as an example, the gap voltage (*V*_gap_) between the top electrode and the bottom triboelectric material is given by Equation 15:^[^ ^221^, ^222^ ^]^

(15) $$V_{\mathrm{gap}}=\frac{t\sigma d}{\varepsilon_{0}\left(t+d\varepsilon_{r}\right)}$$

where *σ* is the triboelectric surface charge density, *t* is the thickness of the triboelectric material and *ε_r_* the relative dielectric constant of the triboelectric material, and *ε*_0_ the vacuum dielectric constant (*ε*_0_ ≈ 8.85 × 10^−12^ F m^−1^). To avoid air breakdown, the *V*_gap_ must be smaller than *V_b_* at any operation gap distance.^[^ ^223^ ^]^ Therefore, improving the dielectric constant of triboelectric materials is one of the effective ways to reduce the negative impact of air breakdown on the surface charge density.^[^ ^224^ ^]^ Numerous microcapacitors are formed in the cellulosic triboelectric materials owing to the addition of fillers, facilitating the trapping of more electrons and resulting in a significantly enhanced surface charge density (Figure 8e).^[^ ^219^ ^]^ However, as the filler loading increases, the distance between the filler particles decreases, leading to electron tunneling. In addition, there is a high probability of electrical breakdown at the interface of the triboelectric material and back electrode (Figure 8f), which eventually leads to a low charge density. In particular, when the filler loading exceeds the percolation threshold, the TENG output decreased sharply. This phenomenon is unfavorable for the development of cellulosic triboelectric materials and the practical applications of TENGs. Furthermore, it is a key issue that must be focused on and addressed in the future.

## 4 Strategies for Dielectric Modulation of Cellulosic Triboelectric Materials

In this section, strategic guidance in terms of structure design and filler selection is mainly provided for the dielectric modulation of cellulosic triboelectric materials. In terms of structure, the improvement of triboelectric properties of cellulosic materials by hierarchical, porous, and network structures is analyzed. The difference in dielectric constant caused by different structures is explained by free volume theory. In terms of fillers, the fillers commonly used for dielectric modulation of triboelectric materials are summarized and their differences are compared. It provides a rich space of filler selection for the dielectric modulation of cellulosic triboelectric materials.

### 4.1 Structural Design for Dielectric Modulation of Cellulosic Triboelectric Materials

**Figure 9:**
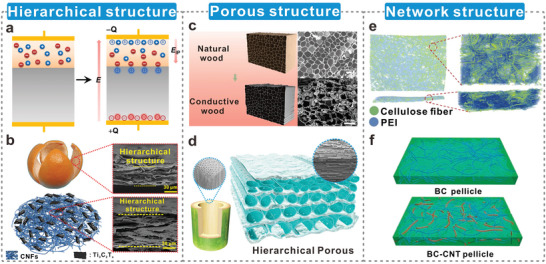
Structural design of cellulosic composite triboelectric materials. a) A novel dielectric film with a hierarchical structure, rich in interfacial regions, induces strong interfacial polarization. Reproduced with permission.^[^ ^226^ ^]^ Copyright 2021, Elsevier. b) Layered cellulosic triboelectric material inspired by the hierarchical structure of grapefruit peel. Reproduced with permission.^[^ ^227^ ^]^ Copyright 2022, Wiley‐VCH. c) A porous conductive wood‐based material loaded with carbon nanotubes, with abundant pores capable of generating additional triboelectric charges. Reproduced with permission.^[^ ^229^ ^]^ Copyright 2021, Elsevier. d) The bamboo‐based cellulosic frictional electric material with a layered porous structure provides a large number of loading sites for PANIs. Reproduced with permission.^[^ ^238^ ^]^ Copyright 2022, Wiley‐VCH. e) The network structure in the cellulose paper facilitates the loading of PEI. Reproduced with permission.^[^ ^239^ ^]^ Copyright 2022, Elsevier. f) The dense network structure of the cellulosic triboelectric material facilitates the dispersion of CNTs. Reproduced with permission.^[^ ^240^ ^]^ Copyright 2021, Elsevier.

The triboelectric properties of cellulose after dielectric modulation can be maximized by designing and optimizing its structure. The design of the layered structure is a common and important strategy in the design of triboelectric materials. It not only increases the surface area of beneficial components in the material but also promotes stronger dielectric polarization.^[^ ^225^ ^]^ Kim et al.^[^ ^226^ ^]^ designed a novel TENG using elastic bilayer films (**Figure** **9**a). The large differences in capacitance and impedance between the bilayer materials cause the migrating charges to be blocked by highly insulating materials, and the charges accumulate at the heterogeneous interface, triggering a strong *P*_int_. Hierarchical cellulosic triboelectric materials inspired by the layered structure of grapefruit peel were recently proposed (Figure 9b).^[^ ^227^ ^]^ Ti_3_C_2_T*_x_* nanosheets and fibrous CNFs are used as “bricks” and “mortars” to form a clear multilayer structure, which increases the interfacial contact area between the MXene filler and the CNF matrix. The potential interface polarization effect will greatly improve the output performance of cellulosic composite triboelectric materials. Moreover, the gas‐sensing properties of MXene also expand the application range of cellulose‐based TENGs. The effect of material porosity on dielectric and triboelectric properties has always been a topic of concern and discussion. In cellulosic materials, the difference in porosity is mainly reflected in the structure of the material, such as flat and smooth films and porous aerogels. Studies have shown that triboelectric materials with porous structures facilitate a larger contact area upon CE and can deform more significantly than dense films, increasing the relative capacitance.^[^ ^228^ ^]^ Considering the fact that the porous structure of natural wood provides carbon nanotubes (CNTs) with several loading sites, Cai et al.^[^ ^229^ ^]^ fabricated a conductive wood‐based triboelectric material (Figure 9c). Not only is the induced charge present on the material surface, but “body charges” are also generated because porous structures can trap additional charges.^[^ ^230^ ^]^ In ion‐rich cellulosic triboelectric materials, the porous structure facilitates the transport of free ions, assists charge carrier migration and enhances ion polarization.^[^ ^231^ ^]^ In addition, abundant pores play a positive role in filler dispersion. Blocking an appropriate amount of nanofiller in each hole can effectively prevent direct contact between the fillers, weaken the threshold effect, and prevent electron tunneling.^[^ ^232^ ^]^ These research data show that the porous structure seems to be very beneficial to the triboelectric performance of the material. However, for the dielectric properties, the contribution of porosity does not seem to be positive. Free volume, defined as the volume not occupied by polymer materials, is considered to be an important factor in determining the dielectric constant.^[^ ^233^ ^]^ The free volume in the form of pores leads to a decrease in the dielectric constant because it is occupied by air with a relative dielectric constant of about 1.^[^ ^234^ ^]^ A higher free volume fraction means that the material will be less dense, resulting in fewer polarizable groups per unit volume. At present, the effect of free volume on the dielectric properties of polymers is still ambiguous.^[^ ^235^ ^]^ The natural network structure inspires the structural design of high‐k cellulosic triboelectric materials.^[^ ^236^ ^]^ The cellulose network structure formed by the interwoven fibers can increase the filler loading.^[^ ^237^ ^]^ Inspired by natural bamboo, Zhao et al.^[^ ^238^ ^]^ prepared a unique bamboo/polyaniline (PANI) triboelectric material with a hierarchical porous structure (Figure 9d). The hierarchical porous structure provides a template for the construction of 3D conductive networks, enhancing the triboelectric properties of the material and enabling it to maintain stable and efficient power output in extreme environments. A polyethyleneimine (PEI)‐loaded paper composite with an extensive fiber network captures and secures much viscous liquid within the composite (Figure 9e).^[^ ^239^ ^]^ Additionally, the synergistic effect of the network structure and functional fillers benefits the physical properties of the material. The network structure of BC in Figure 9f is effectively filled with CNTs, which increases the mechanical and thermal stability of the composite.^[^ ^240^ ^]^ Furthermore, the continuous network structure with strong dipole–dipole interactions facilitates charge hopping from one dipole to another, significantly enhancing the electrical conductivity of the composite.^[^ ^241^ ^]^ The dipole density and charge mobility increase with filler loading, enabling intense dielectric polarization.

### 4.2 Chemical Modification for Dielectric Modulation of Cellulosic Triboelectric Materials

The structural design of cellulosic triboelectric materials is a dielectric modulation strategy based on physical methods, while chemical modification based on the grafting of polar groups is another effective strategy for the enhancement of dielectric constants. For polar polymers including cellulose, the orientation polarizability of the polar groups is a key factor affecting the dielectric constant.^[^ ^242^ ^]^ Each glucose group in the cellulose molecule contains three polar hydroxyl groups. On the one hand, a large number of polar hydroxyl groups make cellulose undergo dipolar polarization under the action of an external electric field, showing a higher dielectric constant than ordinary polymers.^[^ ^243^ ^]^ On the other hand, the active properties of hydroxyl endow cellulose with good chemical reactivity, which in turn makes it a larger space for chemical modification and more possibilities.^[^ ^244^ ^]^ Therefore, the use of higher polar groups to replace the hydroxyl groups on the surface is a good strategy to enhance the dielectric properties of cellulose.

**Figure 10:**
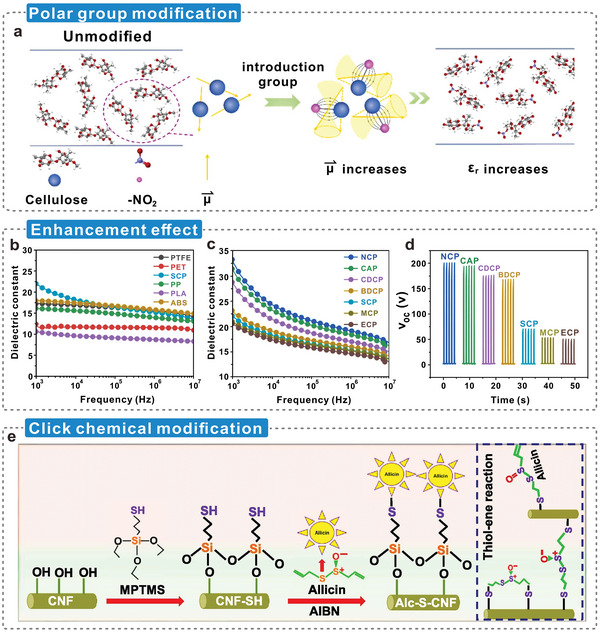
Improving the dielectric properties of cellulosic triboelectric materials via chemical structure modification. a) Schematic diagram of the effect of increasing the dielectric constant of cellulosic triboelectric materials by introducing polar groups/side chains, b) SCP has a higher dielectric constant than common polymer materials, including PTFE, PET, PP, etc. c) These chemically modified SCP papers have a higher dielectric constant than the original SCP, and the dielectric constant of nitrocellulose paper is close to 35, and d) By introducing polar groups, the dielectric constant of cellulose paper is increased, thereby enhancing the triboelectric properties, and the output performance has also been greatly improved. Reproduced with permission.^[^ ^247^ ^]^ Copyright 2022, Elsevier. e) Schematic diagram of the mechanism by which allicin is grafted onto CNF by click chemistry. Reproduced with permission.^[^ ^252^ ^]^ Copyright 2020, Elsevier.

As early as 1977, Shinouda and Hanna^[^ ^245^ ^]^ conducted dielectric measurements on cellulose with different side groups, including cotton cellulose, mercerized cellulose, cellulose acetate, methylcellulose, and carboxymethyl cellulose. The results showed that the values of the dielectric constant, with all cellulose derivatives at a given frequency, are greater than that of cotton cellulose and lower than that of mercerized cellulose. Therefore, they infer that the dielectric behavior of cellulose is closely related to the properties of its side groups. Previous studies have shown that a large increase in the dielectric constant of polymers can be achieved by grafting highly polar groups (such as —CN, —NO_2_, —F), depending on the steering mechanism in the dielectric polarization.^[^ ^246^ ^]^ Li et al.^[^ ^247^ ^]^ grafted different polar groups onto sisal cellulose paper (SCP) by chemical modification, and systematically explored the effects of different polar groups on the dielectric properties and triboelectric output of cellulosic materials. In this study, the polarity of a molecule is determined by the magnitude of its dipole moment. The dipole moment is the product of the distance between the positive and negative charge centers and the amount of charge in the charge center. The greater the dipole moment, the greater the polarity of the molecule.^[^ ^248^ ^]^ The research results show that the molecular dipole moment of SCP grafted with —NO_2_ is the largest, reaching 5.2526 Debye. This is due to the strong polarity of —NO_2_ and its strong electron‐withdrawing ability, which leads to the accumulation of internal charges around —NO_2_ and the increase of the dipole moment.^[^ ^249^ ^]^ As the dipole moment increases, the polarizability and dielectric constant of the cellulosic triboelectric material also increase accordingly (**Figure** **10**a). SCPs used in experiments are primarily composed of sisal fibers. Due to the anisotropy of sisal fibers, the dipoles in the cellulose molecules are aligned along the axis of the cellulose, and the mobility of the dipoles is high, thus showing large polarizability.^[^ ^250^ ^]^ Compared with common polymeric triboelectric materials, SCP shows excellent dielectric properties (Figure 10b). To further improve the dielectric constant of cellulosic triboelectric materials, the authors obtained nitrocellulose paper (NCP), cellulose acetate paper (CAP), methylcellulose paper (MCP), ethylcellulose paper (ECP), chlorodeoxycellulose paper (CDCP), and bromodeoxycellulose paper (BDCP), respectively, by a series of chemical modifications. Among them, the cellulosic triboelectric materials grafted with high dipole moment groups all showed high dielectric constant, and their triboelectric output was correspondingly improved (Figure 10c,d). This is attributed to the fact that the orientation polarization of the grafted strong polar groups weakens the influence of the inherent strong hydrogen bonds of cellulose, thereby improving the dielectric responsiveness.^[^ ^243^ ^]^ The chemical modification method based on group modification has a long research base with mature experience and significant effects, which is worth further exploration and research.

In addition to considering conventional highly polar groups, some unconventional compound molecules have also become strong candidates for the grafting modification of cellulosic triboelectric materials. “Allicin” is an organosulfur or thiocyanate compound found in the garlic clove, consisting of highly dipolar S=O, S—S, and C=C groups.^[^ ^251^ ^]^ The grafting of allicin onto CNFs was achieved by a novel thiol–ene click chemistry. The “click chemistry” was accomplished between the double bonds of allicin and thiol groups which had been pre‐attached to CNFs by a silanization process (Figure 10e).^[^ ^252^ ^]^ Allicin comes directly from fresh garlic juice and is readily available. The modified cellulosic triboelectric material exhibits excellent triboelectric performance, which is about 6.5 times higher than that of the unmodified material and has excellent stability. This all‐green construction concept of high‐performance triboelectric materials could be a valuable addition to scientific research.

Copolymerization of two or more polymers together is a strategy to produce a new material of tailored dielectric properties. In copolymerization, two or more different monomer units were covalently bound thus producing a synergistic effect of respective constituents.^[^ ^233^ ^]^ Copolymerization modification refers to the introduction of functional polymeric side chains on the surface of nanocellulose to change its surface physicochemical properties and endow nanocellulose with new functional properties, which is one of the important methods for functionalization modification of nanocellulose.^[^ ^253^ ^]^ Regrettably, there are few examples of using copolymerization strategies to improve the dielectric properties of cellulose. In the limited research progress, nanocellulose, such as CNC, is often used as an auxiliary component with a small amount of addition,^[^ ^254^ ^]^ and there is no need for a review here. In future research, the copolymerization strategy should be paid attention to in the dielectric modulation of cellulose to fully exploit the chemical activity of cellulose.

### 4.3 Filler Selection for Dielectric Modulation of Cellulosic Triboelectric Materials

**Figure 11:**
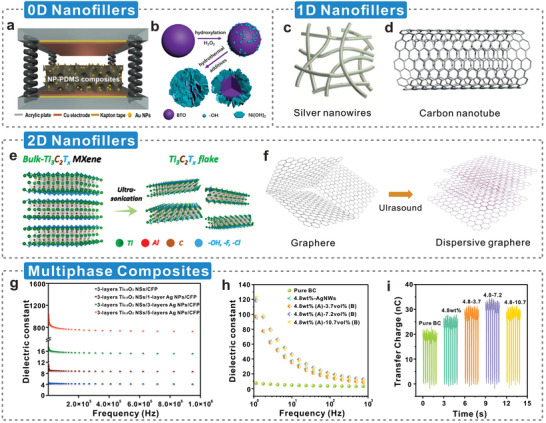
Filler selection of cellulosic composite triboelectric materials. a) Metal‐based conductive nanofillers are used to improve the dielectric constant and output performance of triboelectric materials. Reproduced with permission.^[^ ^256^ ^]^ Copyright 2021, Springer Nature. b) The core–shell filler has a special structure different from ordinary nano‐fillers, which has the effect of lowering the threshold. Reproduced with permission.^[^ ^258^ ^]^ Copyright 2020, Elsevier. c) As the most basic 1D nanofiller, metal nanowires have good dispersion and are often used for dielectric modulation of triboelectric materials. Reproduced with permission.^[^ ^257^ ^]^ Copyright 2019, Wiley‐VCH. d) Carbon nanotubes are used as conductive fillers. Reproduced with permission.^[^ ^273^ ^]^ Copyright 2016, Springer Nature. e) Among 2D materials, MXenes are often used as reinforcing fillers due to their excellent electrical conductivity and attractive layered structure. Reproduced with permission.^[^ ^267^ ^]^ Copyright 2019, American Chemical Society. f) 2D structure of graphene material. Reproduced with permission.^[^ ^268^ ^]^ Copyright 2017, Elsevier. g) Dielectric constants of cellulosic composite triboelectric materials with different numbers of AgNPs impregnated layers. Reproduced with permission.^[^ ^270^ ^]^ Copyright 2020, Wiley‐VCH. h,i) Dielectric constant and transferred charge of cellulosic composite triboelectric materials with different contents of AgNWs and BTO NPs. Reproduced with permission.^[^ ^271^ ^]^ Copyright 2021, Elsevier.

Aside from structural design and chemical modifications, the type and characteristics of the fillers significantly impact the dielectric properties of cellulosic triboelectric materials.^[^ ^255^ ^]^ The fillers are categorized into 0D, 1D, and 2D nanofillers according to their shape and size. Among the spherical 0D particles, metal nanoparticles (**Figure** **11**a) possess high electrical conductivity and dispersibility, resulting in large interfacial regions in the cellulosic insulating matrix and inducing strong *P*_int_.^[^ ^256^ ^]^ Additionally, barium titanate nanoparticles (BTO NPs) in ceramic fillers are commonly used in dielectric modulation owing to their high dielectric constant. The spontaneously polarized ferroelectric domains within BTO NPs generate a localized internal electric field, and randomly oriented dipoles in the polarized state can induce additional charge transfer between the tribolayers.^[^ ^257^ ^]^ As the filler loading increases, the dielectric constant of the composite increases significantly, along with a sharp increase in the loss tangent. The shell layer in the core–shell nanoparticles between the filler and the polymer matrix (Figure 11b) functions as a buffer layer owing to its dielectric constant, weakening the local field strength near the filler, suppressing the leakage current, and reducing dielectric losses.^[^ ^258^ ^]^ Metal nanowires are often added to cellulosic nanocomposites as 1D conductive nanofillers. Silver nanowires (AgNWs) possess excellent electrical conductivity (Figure 11c),^[^ ^259^ ^]^ good economic benefits, and biocompatibility and can be widely used in healthcare systems as well as wearable cellulose‐based TENGs.^[^ ^260^ ^]^ CNTs with a low‐dimensional structure (Figure 11d) possess high charge‐storage capacity, large aspect ratio, and surface area and can absorb heat effectively.^[^ ^261^ ^]^ The successful loading of CNTs can increase the dielectric constant, mechanical strength, and thermal stability and improve the electrical properties of cellulosic triboelectric materials. AgNWs and most carbon materials are minimally harmful to the environment, and the biodegradability of cellulosic triboelectric materials is not compromised by their incorporation. Nevertheless, some eye‐opening research advances now confirm that biomass materials can also be used as a novel dielectric filler for the dielectric modulation of cellulose. Mi et al.^[^ ^262^ ^]^ added silica fibers, human hair, and rabbit hair to CNF aerogels for the first time and achieved a high power generation efficiency. Their study provides a unique approach for the filler selection with respect to high‐performance cellulosic triboelectric materials. Diatom frustules are the siliceous skeleton of aquatic plant diatoms, and the main component is hydrated silica (SiO_2_·*n*H_2_O). For the first time, researchers incorporated it as a biomass filler into cellulosic triboelectric materials, which improved the output performance of CNF‐TENG while maintaining the original biocompatibility and environmental friendliness.^[^ ^263^ ^]^ Therefore, the dielectric constant and sustainability should be considered in the selection process of fillers, which is also an important issue that needs to be paid attention to in future research. Recently, 2D nanofillers have attracted attention owing to their extraordinary physical, electronic, and chemical properties. Among them, carbonaceous materials,^[^ ^264^ ^]^ metal oxide,^[^ ^265^ ^]^ and transition metal dichalcogenides^[^ ^266^ ^]^ are widely used in the dielectric modulation of triboelectric materials. Upon sonication, the 2D‐sheet‐like MXene (Figure 11e)^[^ ^267^ ^]^ and graphene sheet (Figure 11f)^[^ ^268^ ^]^ have good electrical properties and are promising candidate fillers for enhancing the surface charge density. The abundant terminal groups on the surface of MXenes can attract and capture electrons; through an in situ oxidation reaction, they can be converted into high‐k TiO_2_,^[^ ^269^ ^]^ which is an ideal dielectric‐tuning filler. The widespread use of 2D nanofillers is attributed to flake fillers, which easily form a microplate capacitor structure inside the matrix. Furthermore, the small gap between the sheet‐like structures can withstand high breakdown voltages, leading to small dielectric losses.

Compared to two‐phase composites, multiphase composites with three or more components generally have better dielectric properties. Moreover, the additive amount or concentration of each filler component in the multiphase system has a direct impact on the final dielectric properties of the composite. Sriphan et al.^[^ ^270^ ^]^ reported a multiphase cellulosic composite triboelectric material. Cellulose filter paper was used as the substrate and Ti_0.8_O_2_ NSs and Ag NPs were sequentially composited. At a suitable filler ratio, the dielectric constant can reach approximately 800 (Figure 11g). The incorporation of Ti_0.8_O_2_ NSs and AgNPs also leads to additional charged states, further increasing the work function of the composite and facilitating the charge transfer. In this work, Ti_0.8_O_2_ NSs and Ag NPs were added to cellulose filter paper by deposition and impregnation, respectively. Therefore, the amount of filler added can only be measured by counting the number of layers, and it is difficult to give precise guidance. In another related work, the specific effect of the additive amount of Ag NWs and BTO NPs on the dielectric constant of cellulosic triboelectric materials was revealed and the optimal additive amount was published.^[^ ^271^ ^]^ When the content of Ag NWs was fixed at the optimal value of 4.8 wt%, the composites exhibited similar enhanced dielectric constants regardless of adding 10.7 vol% or 7.2 vol% of BTO NPs (Figure 11h). But in terms of triboelectric performance, the cellulosic triboelectric material with BTO NPs content of 7.2vol% showed better electrical output data (Figure 11i). This phenomenon is also attributed to the leakage current due to the percolation threshold and conductive pathways of the composite triboelectric material described earlier.

Blending cellulosic composites with polymers doped with different fillers is another strategy.^[^ ^272^ ^]^ Cellulose–polymer blends offer the advantage of selective filler dispersion, and the percolation phenomenon therein can be reduced more significantly than that in single cellulosic nanocomposites. The remaining studies on dielectric modulation strategies for cellulosic triboelectric materials are summarized in **Table** **4**.

**Table 4:** High‐performance cellulosic triboelectric materials prepared by dielectric modulation

Cellulosic material	Filler selection	Structural design	Electricity output	Potential applications	Refs.
CNF	Phosphorene	Network structure	*V*_OC_: 5.2 V Current density: 1.8 µA m^−2^	–	[274]
Porous CNF aerogel	PEI	Porous structure	*V*_OC_: 106.2 V *I*_SC_:9.2 µA Power density: 13.3 W m^−2^	High‐performance energy harvesting device	[275]
Highly porous CNF composite aerogel	Silica fiber, human hair, and rabbit fur	Porous structure	*V*_OC_: 110 V *I*_SC_:11.3 µA Power density: 3.4 W m^−2^	Ambient mechanical energy harvesting	[262]
Air‐laid paper	Multiwalled carbon nanotubes	Network structure	*V*_OC_: 197 V *I*_SC_:16.2 µA	Self‐powered wearable health monitoring systems	[276]
CMF, CNF	Ag	Hierarchical structure	*V*_OC_: 21.9 V *I*_SC_:0.73 µA Power density: 7.68 µW cm^−2^	Respiratory monitoring	[260]
CNF	Ag, PEI	Gear‐shaped TENG	*V*_OC_: 286 V Power density: 0.43 W m^−2^	High‐efficiency energy harvesting	[277]
Cellulose composite paper	AgNWs, BTO NPs	Network structure	*V*_OC_: 460 V *I*_SC_:23 µA	Large‐area, high‐performance TENG	[257]
Worn‐out cotton textile	PANI for coating	Hierarchical structure	*V*_OC_: 350 V *I*_SC_:45 µA Power density: 11.25 W m^−2^	Wearable electronics	[278]
Commercial printing paper	Polypyrrole (PPy) for coating	Hierarchical structure	*V*_OC_: 60 V Power density: 0.83 W m^−2^	Portable self‐charging power system	[279]
BC nanofibers film	BTO NPs	–	*V*_OC_: 181 V *I*_SC_:21 µA Power density: 4.8 W m^−2^	Wearable electronics	[212]
Cellulose paper	BTO NPs	Porous structure	*V*_OC_: 88 V *I*_SC_:8.3 µA	Wireless transmission system	[280]
Cellulose II aerogel	Chitosan and alginic acid	Porous structure	*V*_OC_: 181 V *Q*_SC_: 92 nC	Green and high‐performance energy harvesting	[87]
CNF	Diatom frustules	Network and porous structure	*V*_OC_: 388 V *I*_SC_:18.6 µA Power density: 18.6 µW cm^−2^	Smart and antibacterial respiratory mask	[263]
Cellulose paper	Nanofumed silica	Hierarchical structure	*V*_OC_: 21.6 V *Q*_SC_: 10 nC	Raindrop energy harvester	[281]
CA	PEI	Porous structure	Power density: 2.21 µW cm^−2^	Ecofriendly TENGs	[282]
Cellulose filter paper	Ti_0.8_O_2_ NSs, Ag NPs	–	*V*_OC_: 42 V Current density: 1 µA cm^−2^ Power density: 25 µW cm^−2^	High‐efficiency energy harvester	[270]
BC	ZnO nanoparticles	Network structure	*V*_OC_: 57.6 V *I*_SC_:5.78 µA Power density: 42 mW m^−2^	Self‐powered implantable and wearable electronics	[283]
CA	Nano‐Al_2_O_3_ fillers	Porous structure	*V*_OC_: 448 V Power density: 2.5 mW m^−2^	Wearable power source	[284]
BC	AgNWs, BTO NPs	–	*V*_OC_: 85 V *I*_SC_:7.1 µA *Q*_SC_: 35 nC	Human–machine interaction	[271]
Cellulose paper	PEI	Network structure	Power density: 79.3 mW m^−2^	Self‐powered pressure sensors	[239]
Wood	CNT	Porous structure	*V*_OC_: 47 V *I*_SC_:2.4 µA *Q*_SC_: 16 nC	Real‐time wireless food‐quality assessment	[229]
Bamboo	PANI	Hierarchical and porous structure	*V*_OC_: 60 V *I*_SC_:2.9 µA Transfer charge density: 174 µC m^−2^	Energy harvesting in extreme environments	[238]

## 5 Dielectric Modulation Endowing Cellulosic Triboelectric Materials with Performance Enhancement

Generally, the combination of proper structural design and functional filler incorporation yields cellulosic materials with excellent triboelectric properties, enabling cellulose‐based TENGs to adapt to multiple environments and actuate in frontier domains such as energy harvesting, wearable electronics, and impedance matching. This section reviews the performance enhancement of cellulosic triboelectric materials after dielectric modulation and envisions their potential for future applications.

### 5.1 Energy‐Harvesting Capabilities Enhancement via Dielectric Modulation

**Figure 12:**
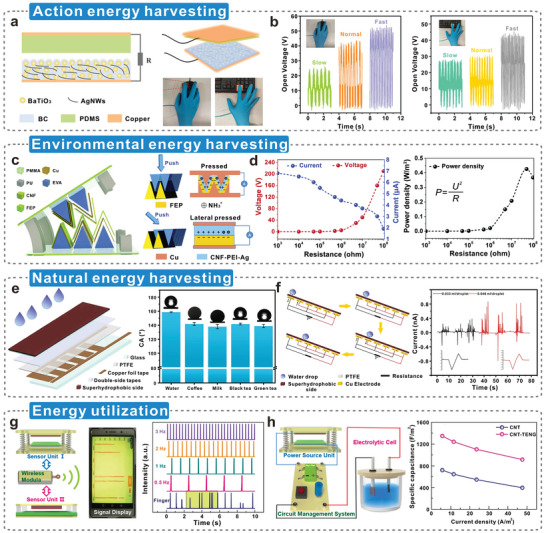
Energy‐harvesting efficiency enhancement via dielectric modulation. a) BC‐based triboelectric materials and TENG structures doped with BTO and AgNWs can generate sensitive electrical output under slight hand movements, b) under different click speeds, the BC‐based composite triboelectric material has an obvious output gradient. Reproduced with permission.^[^ ^271^ ^]^ Copyright 2021, Elsevier. c) Dual promotion of output performance by structural design and dielectric modulation, d) the dual enhancements endow TENG with excellent electrical output performance. Reproduced with permission.^[^ ^277^ ^]^ Copyright 2019, Elsevier. e) A cellulosic composite triboelectric material compounded with MXene and PPy exhibits good hydrophobicity to most liquids, f) Harvesting of liquid energy. Reproduced with permission.^[^ ^287^ ^]^ Copyright 2021, Elsevier. g) TENGs are connected to wireless devices, and the harvested energy is used in smart sensors, h) the energy harvested by TENG is used in the electrolytic cell through the circuit management system to enhance the electrochemical efficiency. Reproduced with permission.^[^ ^280^ ^]^ Copyright 2020, Wiley‐VCH.

Energy harvesting and conversion is one of the most basic and important functions of TENGs. A major purpose of dielectric modulation is to provide cellulosic materials with favorable triboelectric properties, potentializing their application to TENGs for efficient energy harvesting from a wide variety of mechanical energy sources in nature. Yu et al.^[^ ^271^ ^]^ modulated BC with AgNWs and BTO particles and prepared a high‐power‐output BC/AgNWs/BTO‐based TENG that can sensitively harvest energy from human micromotions (**Figure** **12**a). The energy output with microcurrents of 2 and 1.5 µA and a voltage of approximately 25 V were obtained when a mouse and a keyboard embedded with BC/AgNWs/BTO‐TENG (Figure 12b). Moreover, the TENG fabricated with a conductive ferroelectric BC composite paper and BTO could output an electric energy of 460 V and 23 µA, which was sufficient to operate 200 LEDs without any energy storage unit.^[^ ^257^ ^]^ The presence of BTO essentially compensated for the inherent weak electron‐donating tendency of cellulose through forward polarization in the ferroelectric domain. In addition, as a common strategy, the structural design and optimization of TENGs in combination with dielectric modulation of triboelectric materials have been performed to further boost the power output of TENGs.^[^ ^271^ ^]^ Zhang et al.^[^ ^277^ ^]^ designed a gear‐like TENG consisting of a composite cellulosic material with excellent dielectric properties as a positive tribolayer for energy harvesting and sensing applications (Figure 12c). With a suitable number of gears, its maximum output voltage and maximum power density are 210 V and 0.43 W m^−2^, respectively (Figure 12d). Recently, ultrathin noncontact TENGs doped with functional nanofillers have attracted attention owing to their favorable power output and durability without power loss.^[^ ^285^ ^]^ Copper calcium titanate (CCTO) with a high dielectric constant of 7500 is used to improve the output of TENG. Besides the huge dielectric constant, another reason for choosing CCTO is that it combines elements with high electron affinity and the concentration can be easily adjusted by making it into a solution. The introduction of high dielectric particles can effectively improve the dielectric properties and surface roughness of materials. Shao et al.^[^ ^212^ ^]^ found that BC composite films doped with BTO particles had a higher dielectric constant than pure BC films over the entire measurement frequency range, and that the dielectric constant increased with the volume ratio of embedded particles. At the same time, the generation of surface microstructure also has a great effect on the output performance of BC‐based triboelectric materials. These approaches offer a vast outlook for the preparation of cellulose‐based TENGs with advanced power performance.

Adding a high‐dielectric coating on the surface of cellulose is also a common effective method in the dielectric modulation of cellulosic triboelectric materials, and it is also a favorable way to improve the energy harvesting efficiency of cellulose‐based TENG. A high‐dielectric cellulose filter paper coated with PVDF was used as a triboelectric material to develop a green, recyclable, and high‐efficiency energy harvesting device.^[^ ^286^ ^]^ The TENG can rapidly charge a 1 µF capacitor to 15.2 V within 60 s, demonstrating the excellent triboelectric properties of the cellulosic composite filter paper. When dielectric modulation is coupled with hydrophobic modification, cellulose‐based TENGs can harvest energy in humid or even liquid environments. Li et al.^[^ ^287^ ^]^ reported a multifunctional superhydrophobic cellulose composite paper (CCP), whose conductivity and triboelectric properties were significantly enhanced by MXene and PPy (Figure 12e). The CCP‐TENGs maintain the output electricity when the droplets contact and leave regularly (Figure 12f). The excellent energy harvesting capability of superhydrophobic cellulosic composite triboelectric materials is also used in blue energy, and a CNF‐based triboelectric material with silica nanoparticles compounded on the surface has been developed.^[^ ^288^ ^]^ The addition of silica nanoparticles further enhances the hydrophobic properties while forming surface microstructures and enhancing dielectric polarization, enabling the interfacially modified CNF‐based triboelectric material to have stable output performance under humid environments. Besides, the combination of dielectric modulation with other electrical devices will greatly enhance the possibility of cellulose‐based TENG applications. Shi et al.^[^ ^280^ ^]^ fabricated a TENG using cellulose paper coupled with BTO and Ag NPs. The maximum power output of the proposed TENG was 88 V and 8.3 µA, respectively, with a maximum output power of 141 µW at 3 Hz. This advanced cellulose‐based TENG functioned as a sensitive and intelligent motion sensor, remotely monitoring machine operation and delivering messages based on finger movements (Figure 12g). Moreover, the cellulose‐based TENG can drive electrochemical reactions as a smart and portable power source (Figure 12h).

### 5.2 Performance‐Enhanced Cellulose‐Based Wearable Electronic Devices

**Figure 13:**
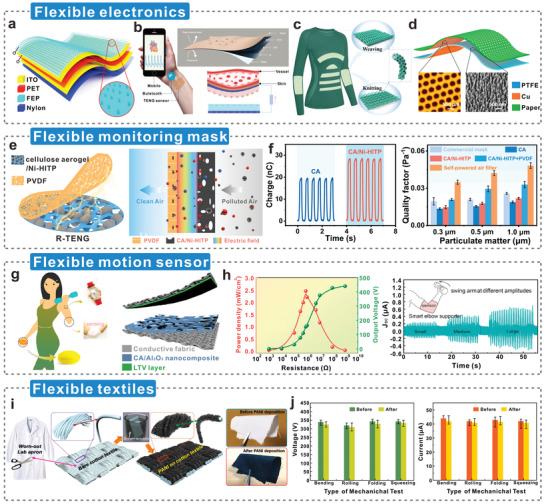
Performance‐enhanced cellulose‐based wearable electronic devices. a) A flexible TENG composed of softer materials for sensitive tactile sensing. Reproduced with permission.^[^ ^290^ ^]^ Copyright 2014, American Chemical Society. b) A low‐cost, lightweight, and durable textile triboelectric sensor for human skin that can be combined with a signal processing module for smart health monitoring. Reproduced with permission.^[^ ^292^ ^]^ Copyright 2021, Wiley‐VCH. c) Smart clothing that enables self‐powered sensing, made with weaving technology. Reproduced with permission.^[^ ^293^ ^]^ Copyright 2022, Wiley‐VCH. d) The first soft and flexible TENG with cellulose paper as the substrate. Reproduced with permission.^[^ ^294^ ^]^ Copyright 2015, American Chemical Society. e) Cellulose‐based flexible self‐powered filter masks and their working principle and f) the incorporation of MOFs leads to a significant improvement in the triboelectric properties of cellulosic triboelectric materials with better filtration and adsorption efficiency. Reproduced with permission.^[^ ^295^ ^]^ Copyright 2022, Elsevier. g,h) Flexible fabric‐based TENG used as a human motion sensor and its working performance. Reproduced with permission.^[^ ^284^ ^]^ Copyright 2020, American Chemical Society. i) The broken cotton fabric loaded with PANI was made into a flexible sensor, and j) Output performance of cotton triboelectric materials before and after compounding PANI. Reproduced with permission.^[^ ^278^ ^]^ Copyright 2019, Elsevier.

Flexibility is an important indicator of triboelectric materials, which affects the development and application of TENGs, especially in wearable electronics.^[^ ^289^ ^]^ In the dielectric modulation of cellulosic triboelectric materials, by doping an appropriate amount of nano‐fillers or compounding with flexible materials, the dielectric properties can be greatly improved to stimulate stronger triboelectric properties, while retaining their own flexibility to the greatest extent. **Figure** **13**a shows a self‐powered ultrasensitive flexible tactile sensor,^[^ ^290^ ^]^ which can be further developed into a complete tactile sensing system upon integration with a signal processing circuit. This flexible tactile sensor inspired the application of TENGs to human‐machine interfaces, skin‐like electronics, industrial automation, medical procedures, and security systems.^[^ ^291^ ^]^ Fang et al.^[^ ^292^ ^]^ developed a low‐cost, lightweight, and durable textile triboelectric sensor (Figure 13b). The sensor realizes intelligent monitoring of human health data when integrated with a wearable signal processing circuit, Bluetooth transmission module, and customized mobile APP. Regarding flexible clothing, TENGs composed of wear‐resistant flexible materials can be combined with traditional clothing textiles to fabricate smart fabrics for human motion detection (Figure 13c).^[^ ^293^ ^]^ This application offers a new prospect for flexible electronic products. Highly flexible cellulosic materials were applied to TENGs first in 2015 (Figure 13d)^[^ ^294^ ^]^ and have aroused extensive research interest since then. Following this, flexible and durable cellulosic triboelectric materials have exhibited great potential in energy conversion, self‐powered devices, flexible wearable sensing systems, human‐computer interaction, and soft robotics applications. Recently, a cellulosic triboelectric material with excellent air permeability and filtering ability was proposed and applied in a flexible self‐powered breathing mask (Figure 13e).^[^ ^295^ ^]^ The addition of metal‐organic frameworks (MOFs) not only enhanced the output performance of the R‐TENG but also endowed the cellulosic triboelectric material with excellent electrical conductivity. Cellulose aerogel (CA)/ conductive MOF (Ni‐HITP) composite material can simultaneously play three roles as a filter, electrode, and triboelectric material. Compared with plain CA, the transferred charge of CA/Ni‐HITP increased from 20 to 28 nC (Figure 13f). This enables R‐TENG to continuously and efficiently provide electrostatic charges to the filter and maintain effective filtration of submicron particles for a long time. Exciting work efficiency benefits from traditional filtration mechanism combined with enhanced electrostatic adsorption mechanism. Similarly, Rajabi‐Abhari et al.^[^ ^263^ ^]^ presented a self‐powered smart flexible face mask for human respiration monitoring, in which the core component (i.e., respiration sensor) is composed of cellulose‐based TENG. CNFs doped with diatom frustules (DFs) are positive triboelectric materials for TENG, and their surfaces are coated with AgNWs. Due to the antimicrobial properties of AgNWs, the face mask was able to monitor human health status as well as against germs. The working mechanism of this smart mask is to use breathing airflow to promote regular contact and separation between tribolayers thus generating periodic output of the electrical signal. Compared with the single‐TENG mask, the dual‐TENG‐based respiration sensor has better performance in monitoring human respiration.

Electrostatic spinning is a popular technology in flexible fiber materials and wearable devices and is often used in the preparation of cellulosic triboelectric materials. A simple and scalable one‐pot electrospinning fabrication technique was utilized to construct all‐fiber‐structured TENGs, in which the positive triboelectric material consisted of ethyl cellulose and polyamide 6 co‐electrospun.^[^ ^296^ ^]^ The introduction of polyamide 6 not only improves the triboelectric properties of cellulosic composite triboelectric materials but also greatly improves the mechanical properties, which is very desirable for wearable electronic devices. Bai et al.^[^ ^284^ ^]^ prepared an environmentally friendly porous nanocomposite fabric‐based TENG by incorporating Al_2_O_3_ nanofillers into the CA networks (Figure 13g). The TENG had a high output power, with a maximum open‐circuit voltage of 448 V and an instantaneous power density of 2.5 mW cm^−2^ (Figure 13h), which is sufficient to monitor human exercise movements in real time. Moreover, owing to the porous structure of CA and even filler dispersion, the fabricated TENG exhibited excellent wash fastness, high mechanical flexibility, and durability. Aside from filler doping, conductive‐polymer coating and deposition on the material surface are alternative approaches for dielectric modulation.^[^ ^297^ ^]^ Uzun et al.^[^ ^298^ ^]^ prepared a knittable and washable multifunctional MXene‐coated cellulose yarn using a traditional method. In their study, conventional cellulose‐based yarns were transformed into highly conductive and electrochemically active fibers and yarns, providing a platform technology for developing various types of textile‐based devices. Dudem et al.^[^ ^278^ ^]^ presented a flexible wearable TENG based on the in situ polymerization of PANI in worn cotton textiles (Figure 13i). Owing to the flexibility and multinetwork structure of cotton textiles, the conductive polymer PANI was intended to strongly adsorb onto the cotton microfibers; consequently, the as‐prepared cotton textiles exhibited excellent electrical performance even after 2000 bendings, rolling, folding, and extrusion cycles (Figure 13j). Dielectric modulation with appropriate nanofillers and conductive polymers not only enhances the triboelectric properties of cellulosic materials but also retains their original flexibility. Considering their original features such as biocompatibility and degradability, the application of biodegradable or recyclable cellulose‐based TENGs is anticipated in the future.

### 5.3 Impedance Matching of TENG Circuits via Dielectric Modulation

**Figure 14:**
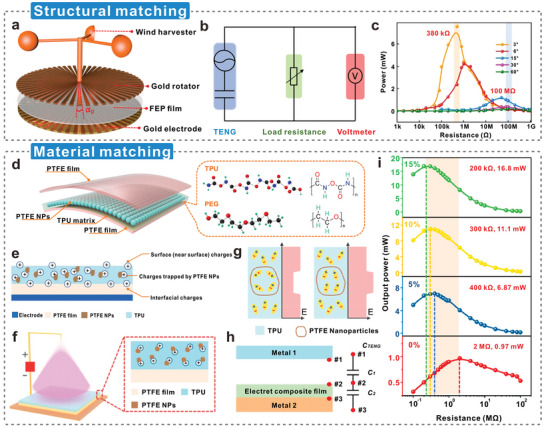
Impedance matching of TENG circuits via dielectric modulation. a) The impedance tunable pinwheel TENG adjusts the internal impedance via changing the device structure, b) the interior of TENG with tunable impedance can be modeled as a capacitor connected in series with an ideal voltage source, and c) the tunable impedance TENG is capable of reducing impedance from a maximum of 100 MΩ to 380 kΩ. Reproduced with permission.^[^ ^301^ ^]^ Copyright 2021, Elsevier. d) Electret composite film with a tunable dielectric constant, e) the addition of short‐chain additives weakens the interaction of molecular chains in the matrix material and facilitates the migration of polarized groups, f,g) A large amount of interfacial area is created between the matrix layer and the nanofiller, h) the principle of impedance matching by increasing the dielectric constant of the material, and i) the optimal matching impedance of TENG can be reduced by about 90% through the strategy of material optimization. Reproduced with permission.^[^ ^302^ ^]^ Copyright 2021, American Chemical Society.

The excellent energy conversion capability of high‐k cellulose‐based TENGs broadens their application prospects in IoT and big data sensing. However, the low power density and high internal impedance of TENGs hinder the large‐scale commercialization of cellulosic triboelectric materials.^[^ ^299^ ^]^ The megohm‐level intrinsic impedance of TENGs is much larger than that of general commercial electronic equipment, which leads to an impedance mismatch of the circuit and unsatisfactory power output.^[^ ^300^ ^]^ To solve this problem, an impedance‐tunable pinwheel TENG was developed, shown in **Figure** **14**a.^[^ ^301^ ^]^ It can be simulated as a capacitor in series with an ideal voltage source (Figure 14b). The maximum internal impedance of the TENGs was reduced from 100 MΩ to 380 kΩ by altering the device structure, thereby changing the signal frequency (Figure 14c). However, the structural design of this TENG is complex and its adjustment and operation are difficult and cumbersome. Furthermore, the device accuracy decreases after long‐term operation.

Wang et al.^[^ ^302^ ^]^ proposed a simple and efficient material optimization strategy that can reduce the matching impedance while improving the output performance of the TENG. As shown in Figure 14d, an impedance‐tunable TENG was fabricated using an electret composite film with a tunable dielectric constant by doping a conductive additive. The hydrogen bonds within the matrix material are controlled by the ionic conductivity of the conductive additives.^[^ ^303^ ^]^ Additionally, short‐chain additives weaken the interaction of the molecular chains within the matrix material (Figure 14e) and promote the migration of polarized groups, increasing the dielectric constant of the composite film. Charge injection is another method to regulate the surface charge density of materials via corona charging. Usually, the injected charges are stored on or near the surface of the matrix composite layers or the interfacial region between the matrix layers and the nanofillers (Figure 14f,g).^[^ ^304^ ^]^ If the dielectric constant of the matrix is large, many nanoparticles will be concentrated in the electric field to capture additional charge in the interface region. Conversely, if the dielectric constant of the matrix is small, few charges will be transferred and captured because of the decrease in potential. Therefore, a material matrix with a suitable dielectric constant is crucial for injecting high‐density charges into the surface of the electret composite film during corona charging treatment to further enhance the charge capture capability and power performance of TENGs. The investigation of the effect of TENG impedance implies that the TENG circuit must be considered as a series of two capacitors, *C*_1_ and *C*_2_ (Figure 14h), where *C*_1_ represents the capacitance of the air layer and *C*_2_ represents the capacitance of the electret composite layer. The equivalent capacitance (*C_TENG_*) and original impedance (*Z*) of the TENG are given by Equations 16 and 17, respectively.^[^ ^305^ ^]^

(16) $$C_{\mathrm{TENG}}=\frac{s\varepsilon_{0}d_{1}\varepsilon_{r}^{2}}{d_{2}^{2}+2\varepsilon_{r}d_{1}d_{2}}$$

(17) $$Z=\frac{1}{j\omega C_{\mathrm{TENG}}}$$

As shown above, an increase in the dielectric constant (*ε*_r_) leads to an increase in *C*_TENG_, thereby reducing the intrinsic impedance of the TENG. Subsequently, the effects of different conductive additive contents on the power performance and internal impedance of the TENG under different external loads were studied. With an increase in the additive content, the maximum power output of the TENG increased sharply by 17.3 times, whereas the optimal matching impedance decreased by 90% from 2 MΩ to 200 kΩ (Figure 14i). The enhanced dielectric constant could reduce the intrinsic impedance of the TENGs and accumulate surface charges of the cellulosic triboelectric material, thereby combinedly boosting the power output of the cellulose‐based TENGs. This coupled enhancement is noteworthy and is a promising development path for high‐k cellulosic triboelectric materials.

## 6 Summary and Challenges

Cellulose, a polymer commonly found in nature, has unique advantages such as natural positive triboelectric properties, high abundance, high flexibility, high chemical reactivity, assemblability, biodegradability, and biocompatibility.^[^ ^306^ ^]^ It has been widely used in IoT applications such as energy harvesting, self‐powered sensors, human‐computer interaction, intelligent monitoring, and flexible wearable electronic devices.^[^ ^307^ ^]^ Cellulosic materials are capable of continuous scale‐up and manufacturing in industries, potentially catering to the substantial demand for future TENGs for sustainable advanced triboelectric materials.^[^ ^308^ ^]^

This review introduces advanced cellulosic triboelectric materials, which are efficient and multifunctional sustainable triboelectric materials fabricated by dielectric modulation. As a promising approach, dielectric modulation greatly expands the application potential of cellulose‐based TENG by polishing cellulosic triboelectric materials. However, the difficulties and challenges presented herein should be focused on and addressed toward ensuring the widespread application of cellulosic triboelectric materials.

### 6.1 Leveraging the Unique Advantages of Cellulose

At present, research on cellulosic triboelectric materials has made significant progress. However, the practical application of cellulose is limited; moreover, the natural advantages and unique properties of cellulose have not been completely harnessed. Cellulose has excellent mechanical flexibility, good biocompatibility, and processability, which are cumulatively the most significant advantage of cellulosic triboelectric materials. Aside from dielectric modulation, chemical modification has proven to be an effective method for increasing the surface charge density in cellulose. The combination of both methods would yield extraordinary cellulosic materials with exceptional triboelectric properties.

### 6.2 Fundamental Research Focusing on the Polarization Properties of Cellulose

In the current research progress, some research teams have done a lot of exciting work on the polarizability and triboelectric properties of cellulosic materials represented by wood. Unfortunately, the polarization properties and mechanisms inside cellulosic materials have not yet been revealed. Studies have shown that cellulose is rich in polar groups and dipoles and has an obvious chain‐like structure. In theory, the dipole moment can be improved by suitable means to create a strong dipole polarization. But in fact, the hydroxyl group is a “double‐edged sword” for the dielectric properties of cellulose. On the one hand, a large number of polar hydroxyl groups in the molecular chain endow cellulose with a larger dipole moment and higher dielectric constant than ordinary polymers. On the other hand, the strong hydrogen‐bonding network based on hydroxyl groups limits the further dipolar polarization of cellulose. In the future, attention should be paid to solving the balance between the two. In addition to dielectric polarization, using a strong electric field to induce dipole rearrangement will also improve the polarization responsiveness of the material, improving the dielectric properties while avoiding high dielectric losses. Recently, the self‐polarization phenomenon of some composites with ferroelectric effect has been widely reported. This mechanism combines the dielectric polarization and self‐polarization of the material itself to significantly improve triboelectric performance synergistically. These creative dielectric control methods are very beneficial for the future application of cellulosic triboelectric materials and the development of cellulose‐based TENGs.

### 6.3 Development of Potential Strategies for Dielectric Modulation of Cellulose

Among the currently available dielectric modulation strategies for cellulose, three main aspects are included, respectively, physical structure design, chemical modification, and filler selection. These strategies have played an important role in the current research progress for the dielectric enhancement of cellulose, but there are still some potentially viable strategies that remain unexplored. In terms of physical structure design, adding suitable dielectric layers or functional layers to triboelectric materials is also an important method to improve the dielectric properties of composites. The ability of triboelectric materials to capture and store charges can be improved through proper structural design, and this method has more reliable controllability. The chemical modification strategy focuses on the replacement of hydroxyl groups in cellulose by highly polar groups with larger dipole moments to obtain higher dielectric response properties. In the present study, cellulose molecules grafted with —NO_2_ have been measured to have a large dipole moment. More highly polar groups should be tried in the future and better simulation of the dipole moment of cellulose molecules should be carried out by software. The hindrance of the degree of polymerization to the difficulty of simulation should be actively addressed in this process. The most noteworthy aspect of filler selection is the aggregation of fillers, which will not only cause the permeation effect but also affect the surface roughness of the material, thus reducing the contact and working efficiency of TENGs. Among them, the interface between nanofillers and polymer matrix is very critical, and surface modification of nanoparticles and selection of suitable core–shell fillers are promising approaches. It is worth noting that due to the complexity of the interaction between the dielectric filler and the polymer matrix, the dielectric constant of cellulosic composite triboelectric materials cannot be accurately predicted with existing theoretical models. Future research should also focus on theoretical models.

### 6.4 Customized Design of Diversified Cellulosic Triboelectric Materials in the Future

In the development of advanced cellulosic triboelectric materials, the closeness of the connection between application scenarios and material design has always been the focus of attention. There are two mainstream ideas for the preparation of cellulosic materials: the “top‐down” strategy, which uses wood and other bulk natural resources as raw materials, and the “bottom‐up” strategy, which uses nanocellulose as a starting point. From 0D to 3D, cellulose has a material selection space with different dimensions and morphologies. Therefore, for different application scenarios and performance requirements, the corresponding preparation process should be selected to obtain customized cellulosic triboelectric materials. For example, several advances have been made and are coming into view for cellulosic gas‐sensitive triboelectric materials using wood and bamboo as raw materials or templates. For cellulosic aerogels or thin films carrying 2D fillers, the hierarchical structure and porous structure are their greatest advantages. The unique natural structure allows the cellulosic gas‐sensitive triboelectric material to establish more gas capture sites, greatly improving the sensitivity of gas sensing. Using nanocellulose as a starting point is a major strategy for stretchable cellulosic materials in human skin and flexible tactile sensing, mainly for cellulosic gels or elastomers. In recent years, triboelectric textiles for self‐powered smart monitoring have been favored due to their good air permeability, safety, and stability. Cellulosic triboelectric fibers made from natural cotton fibers or hemp fibers are strong candidates. For different application scenarios, the most suitable preparation process of cellulosic triboelectric materials should be selected to make different material forms to serve human production and life.

### 6.5 Balance between Triboelectric Properties and Environmental Friendliness of Cellulosic Materials

Cellulose has a natural advantage over common polymeric triboelectric materials in that it is environmentally friendly. To cope with the environmental burden caused by petroleum‐based polymers, renewable, degradable, and recyclable cellulosic triboelectric materials provide a solution for the sustainable development of energy. Therefore, while considering the improvement of the triboelectric properties of cellulosic materials, the focus should be on their environmental adaptability. In previous studies, some toxic fillers were added to cellulosic materials for better electrical output performance, including polyaniline and liquid metal. These materials will not only burden the environment, but also endanger human health, and do not have the potential for sustainable development. When preparing cellulose composite triboelectric materials, degradable and low‐cytotoxic fillers, such as Au, Ag, etc., and biomass fillers such as diatom frustule, should be preferred. In particular, researchers have found that some common biomass wastes, such as human hair and rabbit fur, can also be used as fillers to improve the triboelectric properties of cellulose. Therefore, future research should investigate the effect of different types of fillers on improving the dielectric and triboelectric properties of cellulose, especially environmentally friendly biomass fillers. Cellulose has excellent chemical reactivity, which makes the dielectric modulation strategy based on chemical modification have great development potential. The disadvantage is that the drugs and raw materials involved in some chemical reactions are very harmful to the environment and human body, and the repair cost is high, so they cannot be widely promoted. In the future, the drawbacks of existing chemical modification methods in terms of environmental protection should be actively addressed, and efforts should be made to develop new methods that are greener and lower in energy consumption. More efforts should be made for the industrial production and wide‐scale application of green and sustainable triboelectric materials.

### 6.6 Exploring the Application Prospects of Advanced Cellulose‐Based TENGs

High‐k materials not only contribute to an increased surface charge density but also reduce the internal impedance of the TENGs, combinedly with enhancing the power output of TENGs. Moreover, various fillers endow cellulosic triboelectric materials with additional functions such as electrical conductivity, hydrophobicity, and thermal conductivity. These properties will enable the application of cellulosic triboelectric materials to highly complex and harsh environments and tap into the application prospects of advanced cellulose‐based TENGs. For example, high‐k cellulosic triboelectric materials have broad application value in high‐performance sensor networks, artificial muscles, biomimetic intelligent robots, and microwave devices. At the same time, the hazards associated with high dielectric constants should be carefully attended to when it comes to applications such as instrumentation and communication, military defense, and petrochemicals.

## Conflict of Interest

The authors declare no conflict of interest.
